# Arginyltransferase 1 modulates p62-driven autophagy via mTORC1/AMPk signaling

**DOI:** 10.1186/s12964-024-01499-9

**Published:** 2024-01-31

**Authors:** Laura V. Bonnet, Anabela Palandri, Jesica B. Flores-Martin, Marta E. Hallak

**Affiliations:** 1 Departamento de Química Biológica Ranwel Caputto, Universidad Nacional de Córdoba, Córdoba, Argentina; 2 Consejo Nacional de Investigaciones Científicas y Técnicas (CONICET), CIQUIBIC, Córdoba, Argentina

**Keywords:** Arginylation, Arginyltransferase 1, Autophagy, mTORC1, Posttranslational modification, p62/SQSTM1

## Abstract

**Background:**

Arginyltransferase (Ate1) orchestrates posttranslational protein arginylation, a pivotal regulator of cellular proteolytic processes. In eukaryotic cells, two interconnected systems—the ubiquitin proteasome system (UPS) and macroautophagy—mediate proteolysis and cooperate to maintain quality protein control and cellular homeostasis. Previous studies have shown that N-terminal arginylation facilitates protein degradation through the UPS. Dysregulation of this machinery triggers p62-mediated autophagy to ensure proper substrate processing. Nevertheless, how Ate1 operates through this intricate mechanism remains elusive.

**Methods:**

We investigated Ate1 subcellular distribution through confocal microscopy and biochemical assays using cells transiently or stably expressing either endogenous Ate1 or a GFP-tagged Ate1 isoform transfected in CHO-K1 or MEFs, respectively. To assess Ate1 and p62-cargo clustering, we analyzed their colocalization and multimerization status by immunofluorescence and nonreducing immunoblotting, respectively. Additionally, we employed *Ate1* KO cells to examine the role of Ate1 in autophagy. *Ate1* KO MEFs cells stably expressing GFP-tagged Ate1-1 isoform were used as a model for phenotype rescue. Autophagy dynamics were evaluated by analyzing LC3B turnover and p62/SQSTM1 levels under both steady-state and serum-starvation conditions, through immunoblotting and immunofluorescence. We determined mTORC1/AMPk activation by assessing mTOR and AMPk phosphorylation through immunoblotting, while mTORC1 lysosomal localization was monitored by confocal microscopy.

**Results:**

Here, we report a multifaceted role for Ate1 in the autophagic process, wherein it clusters with p62, facilitates autophagic clearance, and modulates its signaling. Mechanistically, we found that cell-specific inactivation of *Ate1* elicits overactivation of the mTORC1/AMPk signaling hub that underlies a failure in autophagic flux and subsequent substrate accumulation, which is partially rescued by ectopic expression of Ate1. Statistical significance was assessed using a two-sided unpaired *t* test with a significance threshold set at *P*<0.05.

**Conclusions:**

Our findings uncover a critical housekeeping role of Ate1 in mTORC1/AMPk-regulated autophagy, as a potential therapeutic target related to this pathway, that is dysregulated in many neurodegenerative and cancer diseases.

**Supplementary Information:**

The online version contains supplementary material available at 10.1186/s12964-024-01499-9.

## Introduction

As a common response to changing environmental signals, posttranslational modifications (PTMs) of proteins can modulate cellular functions without *de novo* translation or transcription. Among these, posttranslational arginylation has emerged as a relatively lesser-known player that intricately governs the fate of proteins. Arginylation is catalyzed by the evolutionarily conserved enzyme arginyl-tRNA transferase 1 (Ate1), which covalently adds an arginine moiety to target proteins [1, 2]. In mammals, arginylation occurs on the exposed N-terminal alpha-amino groups of Asp, Glu, and Cys (N-terminal arginylation) and at the carboxyl group on internal Asp and Glu residues of proteins (middle-chain arginylation) [3, 4]. The mouse *Ate1* gene encodes at least four Ate1 isoforms (Ate1-1, -2, -3, and -4) [5, 6], which differ in activity and substrate specificity [4]. However, their distribution and localization have not yet been thoroughly explored. Ate1 mediates posttranslational arginylation of a large family of proteins, including signaling and regulatory proteins, cytoskeletal components, transcription factors, endoplasmic reticulum (ER)-residing and secretory proteins, thereby affecting diverse cell biological processes and normal physiology [7–16]. Indeed, constitutive deletion of mouse *Ate1* is embryonic-lethal due to defective heart development [17], whereas postnatal *Ate1* ablation produces broad phenotypes, including neurological and metabolic abnormalities [18, 19].

Although N-terminal arginylation has been associated with the proteolytic N-degron pathway, which is responsible for targeting proteins for degradation via the UPS [20, 21], recent research has revealed its significance beyond this pathway. Notably, several arginylated substrates, which are metabolically more stable [10, 11, 15, 22, 23], avoid this fate, raising intriguing questions about the broader cellular functions of this PTM. One of the elusive aspects of arginylation lies in its connection to macroautophagy (hereafter autophagy), another essential proteolytic pathway that plays a central role in clearing cellular debris, damaged organelles, and aggregated proteins. In eukaryotic cells, the UPS and autophagy are two interconnected systems responsible for regulating protein degradation [24, 25]. In the UPS, misfolded proteins are marked with poly-ubiquitin chains on lysine residues, subsequently recognized and degraded by the 26S proteasome complex [26]. However, when UPS capacity is exceeded or impaired, cells activate autophagy to prevent the accumulation of poly-ubiquitinated (poly-Ub) proteins and toxic species, thereby maintaining cellular homeostasis [27, 28].

Autophagy is a conserved cellular mechanism that mediates intracellular degradation and recycling of cytoplasmic contents. A basal (or constitutive) level of autophagy is required to maintain cells free of damaged proteins and organelles, while induced (or adaptive) autophagy is a stress-response that promotes cell survival by recycling intracellular components, such as during nutrient starvation [29]. Changes in intracellular nutrient and energy status are mainly conveyed to the autophagic machinery via modulation of mammalian target of rapamycin complex 1 (mTORC1) and AMP-activated protein kinase (AMPk) signaling cascades. In complex cellular networks, signal specificity and crosstalk are crucial for accurate responses to various stimuli. Key to this process are protein scaffolds, such as the autophagic adaptor p62 (also referred to as Sequestosome 1), that assembles specific signaling complexes within different cellular compartments, assuring precise spatial and temporal signaling [30]. p62, which interacts with ubiquitin through the UBA domain and drives selective autophagy via its interaction with microtubule-associated protein 1 light chain 3 (LC3) [31, 32], serves as a functional docking site that recruits components of the mTORC1 signaling machinery to a specific subcellular location [33, 34]. Recent studies have shown that N-terminal arginylated substrates interact with p62 during autophagic removal of the ER and peroxisomes [35–37]. However, the implication of Ate1 in the regulation of this process has not been extensively examined. This study provides a comprehensive analysis of the role of Ate1 enzyme in the autophagy pathway. Herein, we report that Ate1 distribution is coordinated with the p62 adaptor along with UPS substrates, exerting a stimulatory effect on driving selective autophagy. Genetic inhibition of Ate1 impairs autophagic flux, resulting in p62 and autophagic vacuole build-up. Additionally, this research highlights the central participation of Ate1 in the modulation of autophagy in response to nutritional stress, involving the mTORC1/AMPk upstream signaling node, which is responsible for this regulatory effect. These findings emphasize the crucial role of Ate1-mediated arginylation in orchestrating proteolytic mechanisms and maintaining cellular quality control.

## Results

### Proteasomal blockade induces Ate1 perinuclear clustering.

**Fig. 1:**
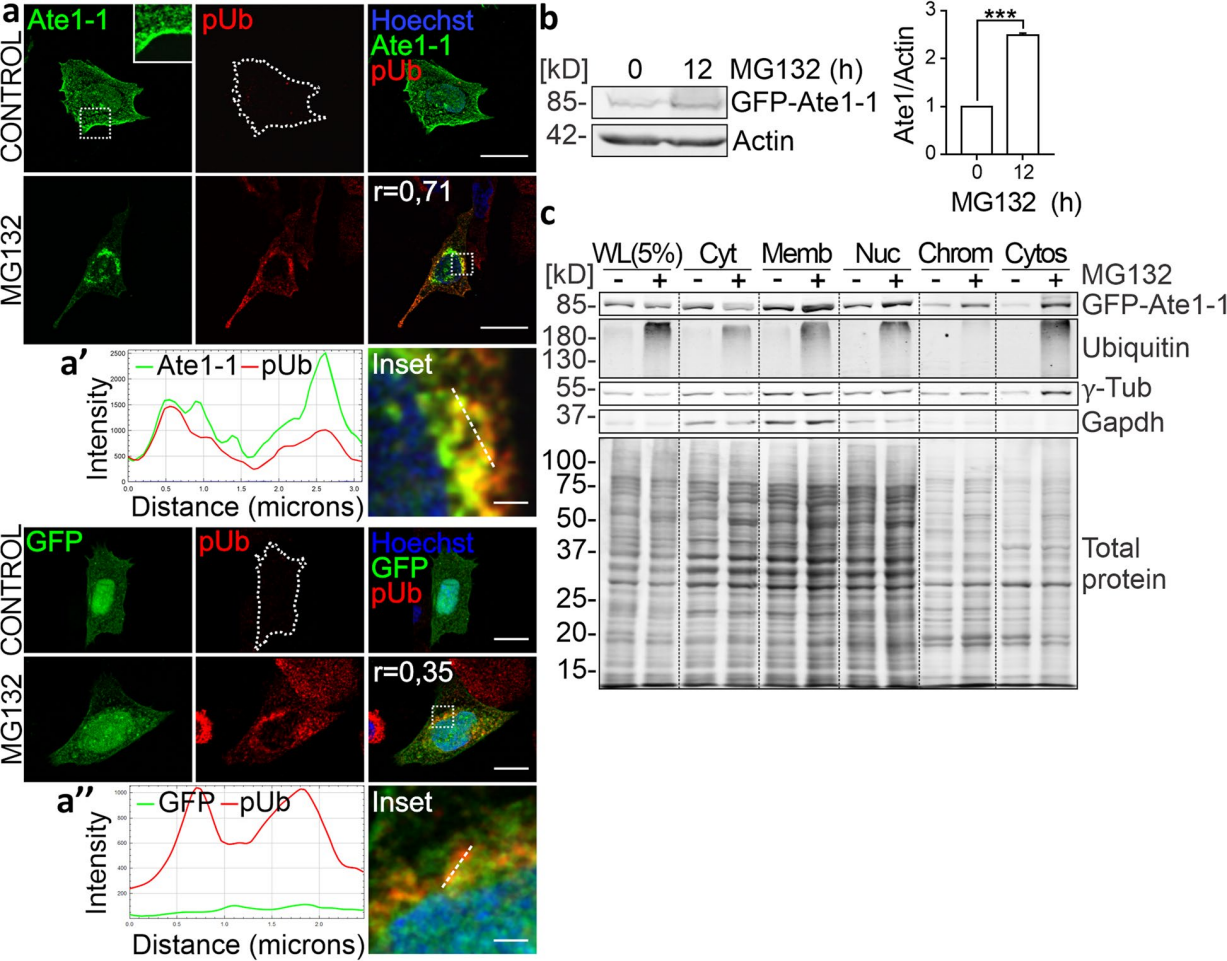
Proteasomal blockade induces Ate1 perinuclear clustering. **a** Immunofluorescence of CHO-K1 cells transfected with pEGFP (GFP) and pEGFP-N2-Ate1-1 (Ate1-1) and incubated with 10 μM MG132 for 12 h. Immunostaining was performed and analyzed using confocal microscopy. Ate1-1 (green) was detected using an antibody against the GFP epitope, and poly-Ub (pUb, red) proteins were identified using a specific antibody that did not cross-react with mono-ubiquitinated proteins. Nuclei (blue) were stained with Hoechst dye. Pearson's correlation coefficient (r) is provided. (**a', a”**) (right panel) Insets show higher magnification of the selected regions (white dashed box). (left panel) Histogram of Ate1-1 or GFP and poly-Ub signal intensity levels along the dashed line indicated in the inset. **b** GFP protein levels in CHO-K1 cells transfected with pEGFP-N2-Ate1-1 (Ate1-1) and treated with 10 μM MG132 (0, 12 h). Ate1-1 protein level was detected using an antibody against the GFP epitope. Actin was used as a loading control. Quantification of Ate1-1 levels at 12 h relative to 0 h is shown. Statistical significance was analysed using two-sided unpaired t-test. Quantifications are representative of two independent experiments and correspond to mean ± SE. ***P≤0.001. **c** *Ate1* KO^Ate1-1^ cells were incubated with 10 μM MG132 for 16 h. Subcellular fractionation was performed using a protein separation kit according to the manufacturer's instructions. Whole lysate (WL) and extracts of cytosolic proteins (Cyt), membrane-associated proteins (Memb) from the ER, Golgi, mitochondrial and plasma membrane, soluble nuclear proteins (Nuc), chromatin-bound proteins (Chrom) and cytoskeleton-associated proteins (Cytos) were obtained. Ate1-1 level were detected using an antibody against Ate1. Gapdh and γ-Tubulin (γ-Tub) were used as markers for cytosolic and cytoskeleton-associated proteins, respectively. The total protein marker was used as a loading control. Scale bars: 10 μm (main images), 1 μm (inset)

As a first step in defining the role of arginylation beyond UPS targeting of arginine-tagged proteins, we characterized the subcellular distribution of Ate1 under proteasomal inhibition. For this purpose, CHO-K1 cells transiently transfected with the green fluorescent protein (GFP)-tagged Ate1-1 isoform or GFP empty construct were incubated with the proteasome inhibitor MG132. Mock- and Ate1-transfected cells were fixed and immunostained for GFP and poly-Ub proteins to control proteasome activity. Confocal microscopy images showed that Ate1-1 localized in the cytosol, nucleus, and near the plasma membrane, whereas poly-Ub proteins were not detectable because they were degraded by the UPS machinery under control conditions (Fig. 1a). Conversely, proteasomal inhibition caused accumulation of poly-Ub proteins and Ate1-1 in aggregated patterns in the perinuclear region of the cytoplasm, which correlated with an upregulation of this isoform, as indicated by the protein expression profile analyzed by western blot (Fig. 1b). Moreover, the Ate1-1 and poly-Ub proteins signals overlapped, as shown by the colorimetric result in merged images (yellow) and the histogram profile (Fig. 1a’). Indeed, Pearson's correlation coefficient (r) indicated a high degree of colocalization. In sharp contrast, mock-transfected cells did not undergo any change in GFP distribution (Fig. 1a, a’’), indicating that proteasomal blockade induces Ate1-1 clustering together with undegraded UPS substrates at the nuclear periphery.

To further determine the cellular compartment in which this enzyme localizes, we performed subcellular fractionation of *Ate1* knockout (KO) mouse embryonic fibroblast (MEFs) cells stably expressing the GFP-tagged Ate1-1 isoform (*Ate1* KO^Ate1-1^). Extracts of cytosolic, membrane-bound (plasma, mitochondria, ER and Golgi), nuclear, chromatin-bound, and cytoskeleton-associated proteins were obtained and analyzed by western blotting. As expected, Ate1-1 was recovered in the cytosolic and nuclear soluble fractions; likewise, a significant proportion of this isoform was detected in the fraction of membrane-bound proteins (Fig. 1c). In response to proteasomal inhibition, the cytosolic level of Ate1-1 decreased, while it was notably enriched in the membrane- and cytoskeleton-bound protein fractions, concomitantly with ubiquitinated proteins. This analysis was also performed using MEFs expressing GFP-tagged Ate1 isoforms 2, 3, and 4 and revealed an expression pattern and treatment response comparable to those observed for Ate1-1 (Fig. S1). These results demonstrate that under proteasomal inhibition, a subset of Ate1 relocalizes to the perinuclear region along with undegraded UPS substrates, where it may further associate with endomembranes and the cytoskeleton.

### Ate1 associates with microsomal membranes under proteasomal inhibition.

**Fig. 2:**
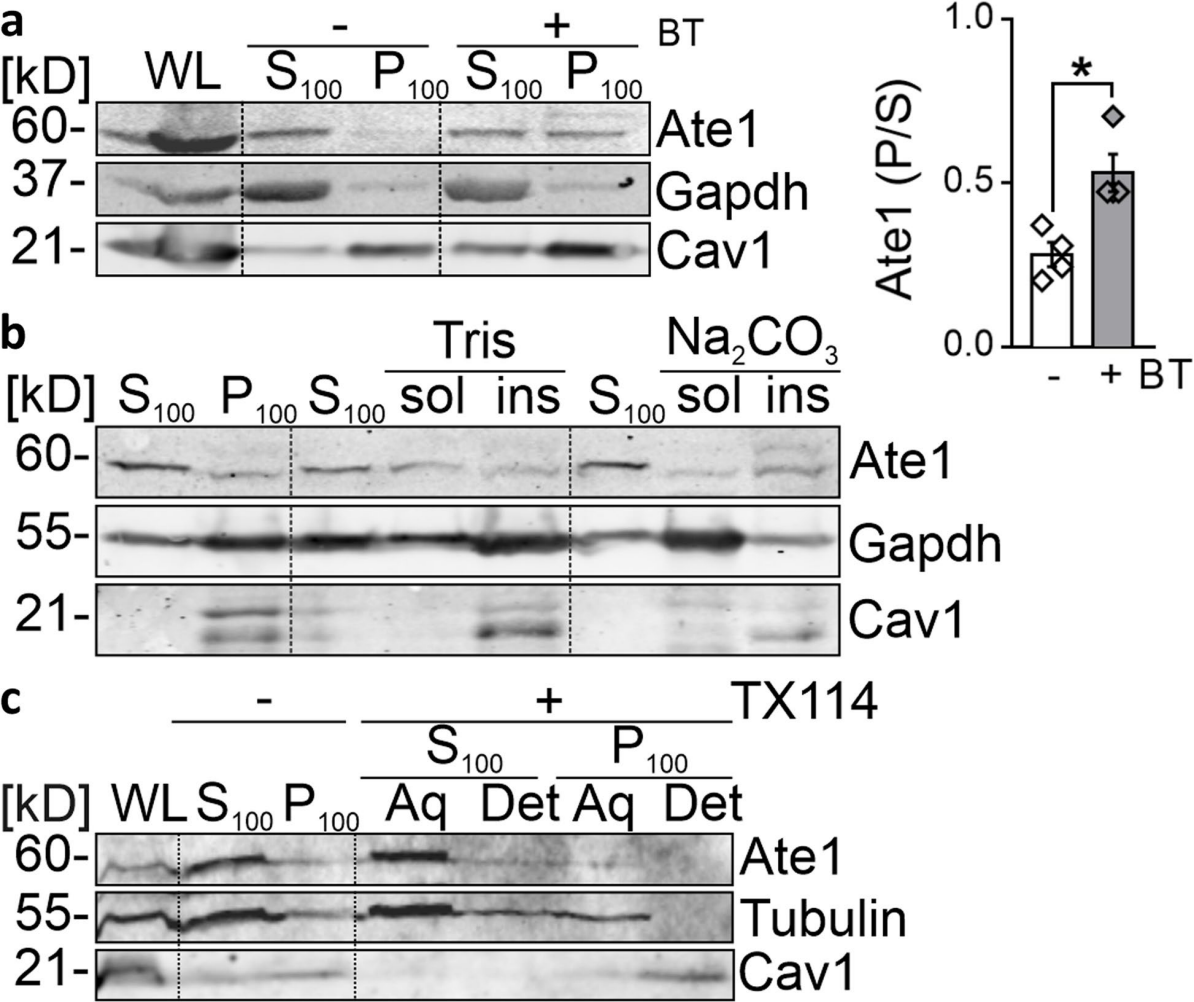
Ate1 associates with microsomal membranes under proteasomal inhibition. **a** Human oligodendroglioma (HOG) cells were treated with 500 nM BT for 16 h. Cell lysates were subjected to ultracentrifugation at 100,000 rpm to separate them into WL, supernatant (S_100_) and precipitate (P_100_) fractions. Cav1 and Gapdh were used as endogenous controls for membrane-bound and soluble proteins, respectively. (right panel) Quantification of Ate1 levels in the P_100_ fraction relative to the S_100_ fraction. Statistical significance was assessed using a two-sided unpaired *t* test. Quantification represents the mean ± SE of four independent experiments. *P≤0.05. **b** Properties of Ate1 association with membranes. The S_100_ and P_100_ fractions from BT-treated HOG cells were prepared as described in (a). The P_100_ fraction was then incubated for 40 min with Tris buffer or 0.1 M Na_2_CO_3_. After treatment, solubilized proteins (sol) were separated from those remaining bound to membranes (ins) by ultracentrifugation at 100,000 rpm and analyzed by Western blotting. Cav1 and Gapdh were used as markers for non-extractable membrane protein and soluble protein, respectively. **c** Detergent partitioning of Ate1 into the S_100_ and P_100_ fractions. The S_100_ and P_100_ fractions from HOG cells treated with BT, obtained by ultracentrifugation at 100,000 rpm, were incubated with TX114 and partitioned into the aqueous phase (aq) or detergent (det). The fractions were subsequently analyzed by Western blotting. Cav1 and Tubulin were used as endogenous controls for the membrane-bound and soluble proteins, respectively. (a-c) Endogenous Ate1 levels were detected using an antibody against Ate1

To facilitate protein degradation at specific locations, cytosolic proteolytic machineries may concentrate in cellular subcompartments associated with membranes [35, 38, 39]. To further determine whether Ate1 is associated with microsomal membranes, HOG cells incubated with the proteasome inhibitor bortezomib (BT) were subjected to subcellular fractionation into soluble (S_100_) and rough particulate cell compartments (P_100_) by ultracentrifugation. Endogenous Ate1 levels were monitored by immunoblotting, and the protein markers Gapdh and Caveolin-1 (Cav1) were run simultaneously as controls for soluble and membrane-associated proteins, respectively. As expected, we observed a twofold increase in Ate1, which was recovered into the cell particulate fraction in response to proteasomal inhibition (Fig. 2a), thereby confirming that a significant pool of the endogenously expressed enzyme is associated with microsomal membranes. Based on this observation, we further characterized the membrane binding properties of Ate1 in proteasome-deficient cells. To this end, the precipitate fraction was subjected to ultracentrifugation after incubation with sodium carbonate at pH 11.5. Exposure of this fraction to alkaline conditions did not result in Ate1 solubilization; in fact, it remained almost completely in the insoluble fraction (Fig. 2b), suggesting that Ate1 clustering is not exclusively driven by electrostatic interactions. To rule out a possible PTM of this enzyme, i.e., lipidation and/or integration into membranes, we performed a Triton X-114 (TX114) partitioning assay on the particulate cell fraction to assess whether Ate1 is solubilized into detergent-soluble (aqueous) and/or -insoluble (detergent) phases. Despite being retrieved into the cell’s particulate compartments, Ate1 was solubilized in the aqueous phase (Fig. 2c), thus revealing that Ate1 behaves as a hydrophilic, soluble protein. In contrast, Cav1 was recovered in the detergent-phase, as expected for a hydrophobic integral protein membrane. Consistent findings were observed in CHO-K1 cell line (Fig. S2). Collectively, these data indicate that a fraction of Ate1 associates with microsomal membranes by a detergent-labile interaction in response to proteasomal blockade.

### Ate1 assembles into p62 bodies.

**Fig. 3:**
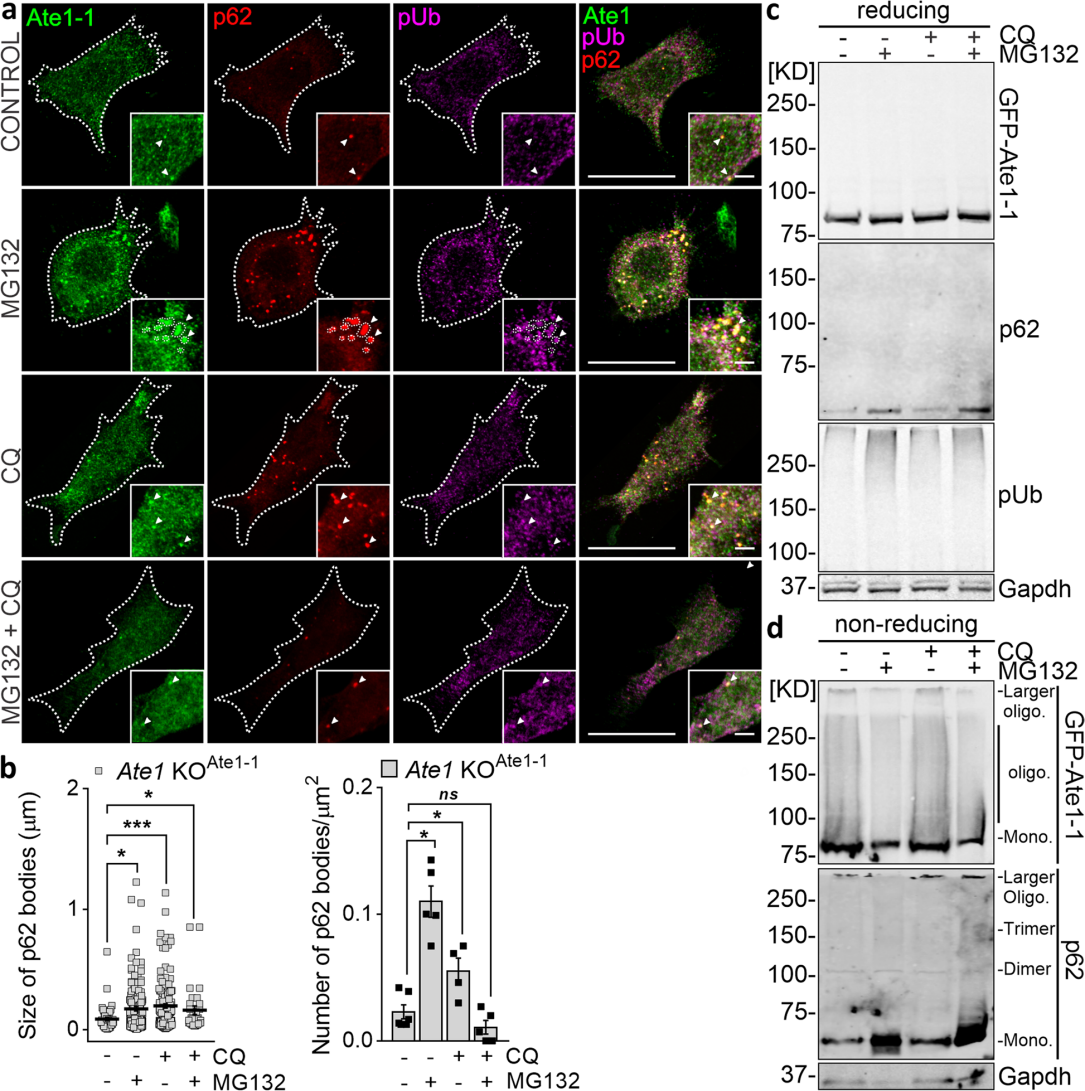
Ate1 assembles into p62 bodies. *Ate1* KO^Ate1-1^ cells were incubated with 10 μM MG132 and/or 20 μM CQ for 6 h (a, b) or 12 h (c, d). **a** Immunostaining was performed using antibodies against Ate1 (green), p62 (red), and poly-Ub (pUb, magenta) and visualized with confocal microscopy. Insets show a higher magnification of puncta where Ate1 and p62 are colocalized (white arrows). **b** Quantification of the size (left panel) and number (right panel) of p62 bodies in *Ate1* KO^Ate1-1^ cells. CONTROL, *n*=50/6; MG132, n=176/6; STV/CQ, *n*=138/5; STV/CQ+MG132, n=16/5 bodies/cells were analyzed. The relative area covered by and the number of p62-labeled inclusion bodies were obtained from maximum intensity projections of confocal z stacks. **c** Immunoblot of Ate1-1, p62 and poly-Ub proteins levels in reducing conditions. **d** Total homogenates of treated cells were separated by SDS-PAGE without β-mercaptoethanol (nonreducing) and western blotted. p62 was used as an endogenous positive control. Oligo.: oligomers, Mono.: monomer. (c, d) Ate1-1 was detected using an antibody against Ate1, with Gapdh as a loading control. Statistical significance was assessed using a two-sided unpaired *t* test. *P≤0.05, ***P≤0.001, *ns*=nonsignificant. Scale bars: 20 μm (main images), 3 μm (inset)

It has been previously established that blockade of the 26S proteasome complex elicits a compensatory p62-driven autophagic response to cope with accumulated unfolded or misfolded protein clearance [32, 40, 41]. To facilitate this process, we speculated that Ate1 and UPS substrate clusters might be spatially close to this autophagic receptor, particularly when proteasome activity is compromised by chemical inhibition in *Ate1* KO^Ate1-1^ cells. To test this hypothesis, confocal microscopy was used to examine the subcellular distribution of p62, which exists as both soluble monomers and insoluble cytoplasmic inclusion bodies (referred to as p62 bodies) [32, 42–44]. Interestingly, Ate1-1-expressing cells exhibited several cytoplasmic p62^+^ puncta, which were spatially correlated with the small foci of this isoform under control conditions (Fig. 3a). Moreover, cells subjected to 6 h of proteasomal inhibition displayed an increased number and size of p62 bodies (Fig. 3b) and a higher level of total p62 after 12 h of treatment, as indicated by the protein expression analysis (Fig. 3c). Importantly, Ate1-1^+^ puncta strongly colocalized with p62 bodies and partially overlapped with poly-Ub proteins, as shown in the merged images (Fig. 3a). Additionally, lysosomal inhibition with chloroquine (CQ), a known autophagy-blocking agent, induced a similar distribution of Ate1-1 and p62. Overall, these data indicate that Ate1 clusters alongside p62, and this assembly appears to occur in control condition and increases in response to proteasome or autophagy inhibition. Similar findings were observed in wild-type (WT) MEFs expressing all four endogenous isoforms (Fig. S3a). Note that within Ate1-1^+^p62^+^ puncta, Ate1-1^+^ puncta were smaller than, and morphologically different from p62^+^ puncta. In contrast, a few Ate1^+^ puncta were spatially correlated with LC3^+^ puncta (Fig. S3b), suggesting that Ate1-1 may initially be targeted to p62 bodies and subsequently delivered to LC3^+^ autophagosomes.

Based on these findings, we next evaluated whether Ate1 forms supramolecular complexes by exploring the possible multimerization status of this enzyme. To do so, we conducted a qualitative analysis through denaturing nonreducing SDS-PAGE of *Ate1* KO^Ate1-1^ cells using p62 as an endogenous positive control for oligomerization. Under both control and proteolytic inhibition conditions, we detected a concentrated band corresponding to the Ate1-1 monomer with an apparent molecular weight (MW) of 85 kD, along with a remarkable smear of multiple oligomer bands (Fig. 3d). Larger oligomers (or aggregates) can be seen on the top of the gel as entities with MW >250 kD. Under reducing conditions, these structures disappeared, and the >250 kD band smeared down to 85 kD, suggesting that any multimers present in the sample were noncovalently bound and/or contained disulfide bonds. In the same experimental settings, p62 appeared as an ~62 kD migrating band and as a homo-oligomeric species. Proteasomal inhibition caused an accumulation of p62 monomers (Fig. 3d) and trimers, as observed in non-heated samples by nonreducing SDS-PAGE (Fig. S3c), whereas autophagy inhibition led to the buildup of larger p62 oligomeric forms. In agreement with this, the inhibition of both proteolytic machineries resulted in the accumulation of monomeric and oligomeric forms. Overall, these results showed that Ate1 might form heteromeric complexes stabilized by disulfide bonds that might be recruited to p62 bodies.

While it is plausible that Ate1 itself may be a substrate of degradation within p62 bodies, half-life assays conducted in the presence of the translation inhibitor cycloheximide (CHX) revealed the conserved stability of Ate1 isoforms, as evidenced by their prolonged half-life (Fig. S3d). This suggests that they are less prone to cellular degradation, allowing them to persist and potentially play a more significant role in cellular proteolytic processes. In summary, this evidence strongly supports our hypothesis that Ate1 clusters, when assembled into p62 bodies, may facilitate the autophagic clearance of undegraded substrates.

### Loss of Ate1 hinders p62 body clearance

**Fig. 4:**
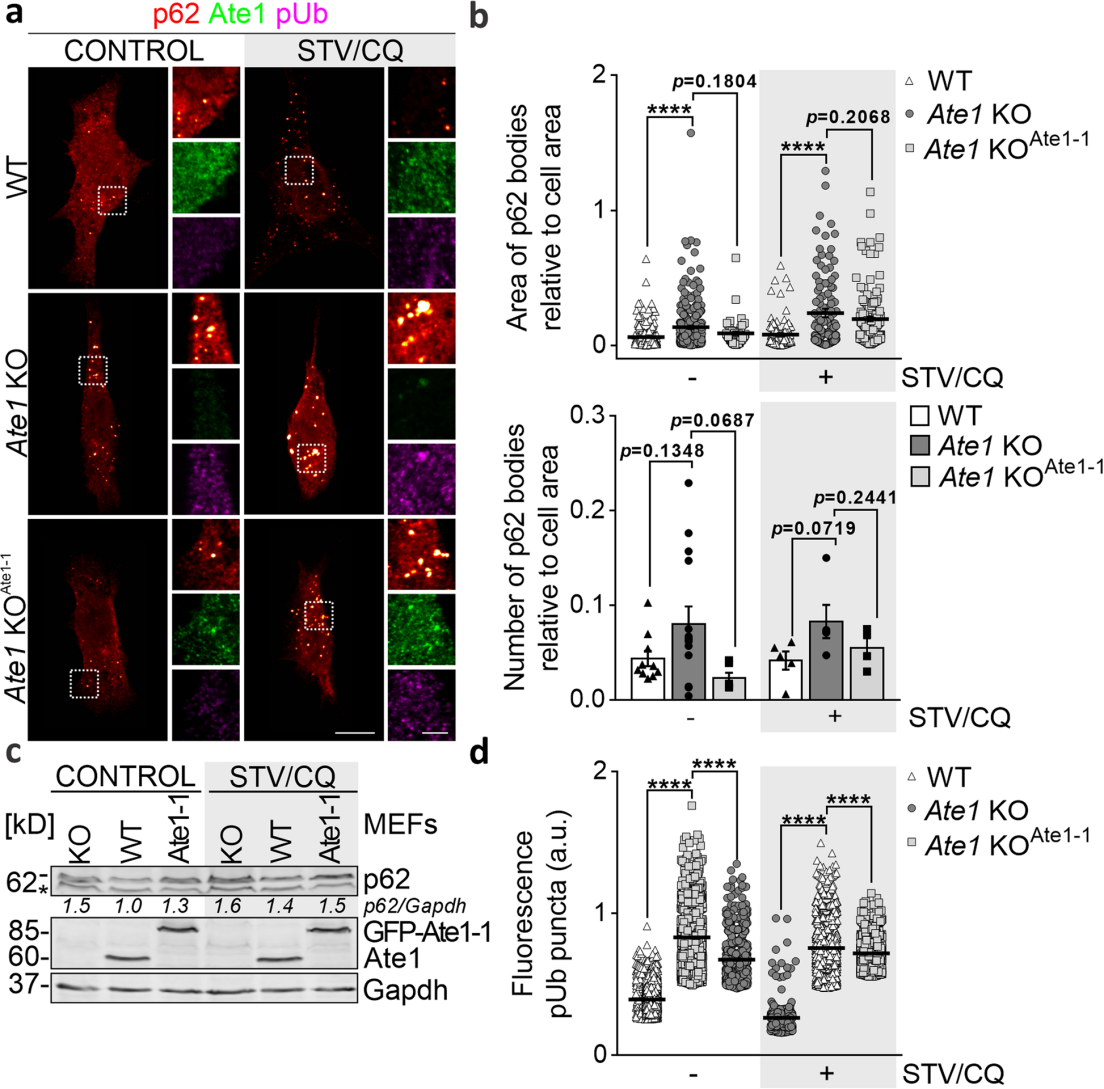
Loss of Ate1 impairs p62 body clearance. WT, *Ate1* KO, and *Ate1* KO^Ate1-1^ cells were cultured in serum-rich (CONTROL) conditions or incubated with 20 μM CQ under STV conditions (STV/CQ) for 6 h. **a** Immunostaining of p62 bodies was performed using p62 (red hot), Ate1 (green), and poly-Ub (pUb, magenta) antibodies and visualized with confocal microscopy. Images are maximum intensity projections of confocal “z” stacks, and insets show single plane images at higher magnification of the selected regions (white dashed box). **b** Quantification of the size (upper panel) and number (lower panel) of p62 bodies, relative to cell area. The relative area covered by and the number of p62-labeled inclusion bodies was quantified using the “Analyze particles” tool of Fiji ImageJ software. CONTROL, *n*=242/337/50; STV/CQ, *n*=112/132/138 bodies were analyzed in WT, *Ate1* KO, and *Ate1* KO^Ate1-1^ cells, respectively. **c** Immunoblot analysis of p62 protein levels. Ate1 and Gapdh were used as *Ate1* deletion and loading controls, respectively. (*) p62 antibody IgG heavy chain. Densitometric analysis was performed, and the relative p62 expression levels are provided below each lane. **d** Quantification of poly-Ub puncta fluorescence intensity. The relative area covered by poly-Ub labeled puncta was quantified using the “Analyze particles” tool of Fiji ImageJ software. CONTROL, *n*=3702/2446/622; STV/CQ, *n*=3768/1360/1377 puncta were analyzed in WT, *Ate1* KO, and *Ate1* KO^Ate1-1^ cells, respectively. Statistical significance was assessed using a two-sided unpaired *t* test. ****P≤0.0001, *ns*=nonsignificant. Scale bars: 10 μm (main images), 3 μm (inset)

To comprehensively elucidate the functional impact of Ate1 on selective autophagy, we carried out a loss-of-function analysis using *Ate1* KO cells. To assess whether p62-mediated clearance is compromised in the absence of Ate1, we first monitored p62 bodies, which are known to be degraded via the autophagy-lysosome pathway [40]. Confocal microscopy analysis revealed a significant increase in the number and relative area of p62-labeled inclusion bodies in *Ate1* KO cells compared to WT cells under both control and serum starvation (STV)/CQ conditions (Fig. 4a, b). Consistent with this, immunoblotting showed higher levels of p62 in the Ate1-deficient cells (Fig. 4c). Because p62 is involved in the lysosomal clearance of undegraded UPS substrates [44], we also assessed poly-Ub proteins levels following disruption of Ate1 and observed an accumulation of poly-Ub^+^ condensates, as indicated by their increased fluorescence intensity (Fig. 4a, d). These data indicate that the loss of Ate1 may compromise p62-cargo complex clearance. Conversely, stable expression of Ate1-1 in the deficient background tends to rescue this phenotype, suggesting that Ate1 is a key component required for p62-driven autophagy.

### Impaired autophagic flux after Ate1 disruption

**Fig. 5:**
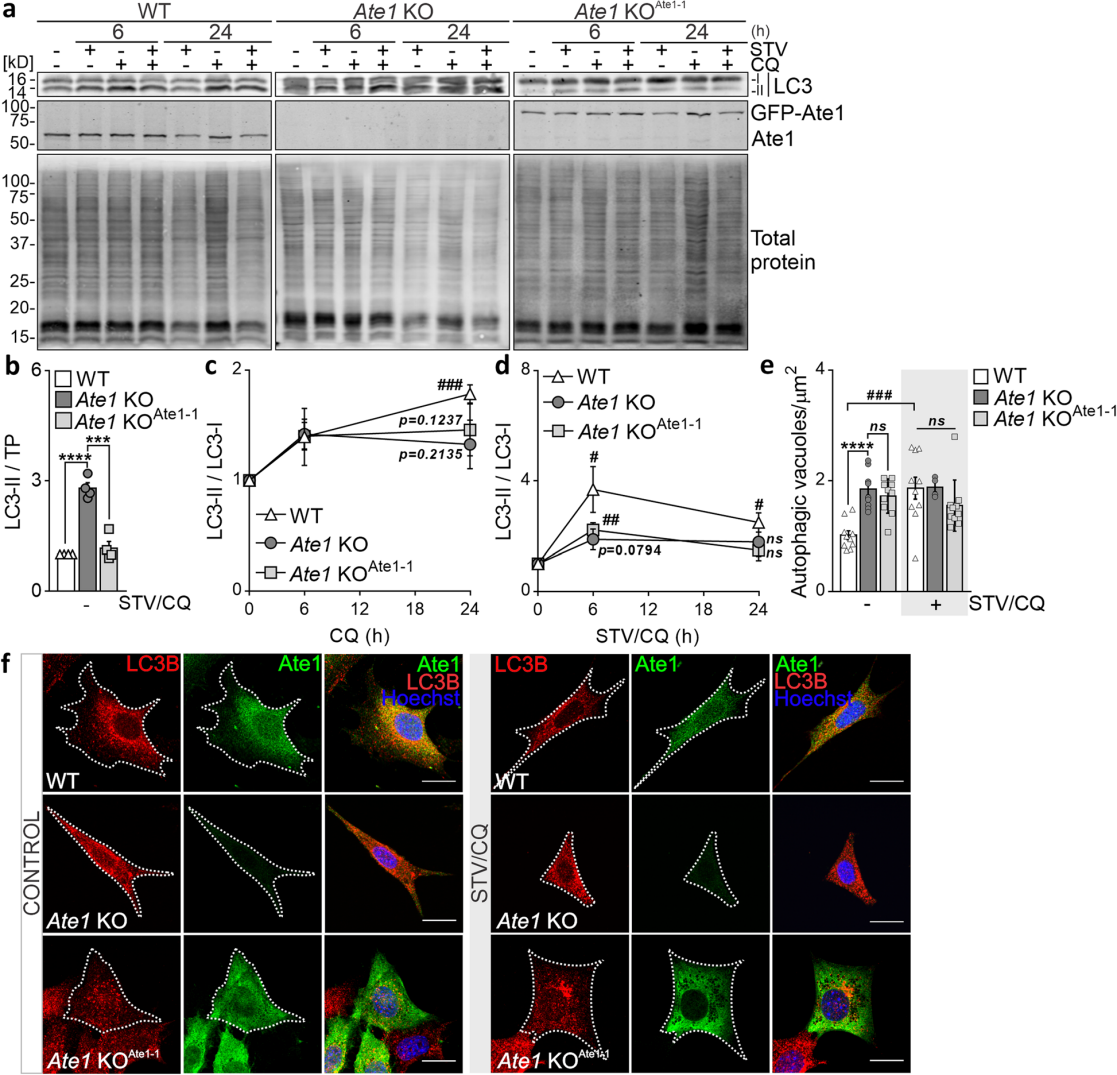
Impaired autophagic flux after Ate1 disruption. WT, *Ate1* KO, and *Ate1* KO^Ate1-1^ cells were treated with 20 μM CQ and/or STV for 6 and 24 h. **a** Immunoblot analysis of LC3B levels. Ate1 and total protein markers were used as *Ate1* deletion and loading controls, respectively. All immunoblot samples were obtained from the same experiment and were processed simultaneously. **b** Quantification of LC3-II relative to total protein marker under serum-rich conditions. Quantification represents the mean ± SE of four independent experiments. **c**, **d** Quantification of the LC3-II/LC3-I ratio under serum-rich (b) or serum-starved (c) conditions. Quantifications represent the mean ± SE of three independent experiments. **e** Immunofluorescence analysis of autophagic vacuoles in WT, *Ate1* KO, and *Ate1* KO^Ate1-1^ cells treated with 20 μM CQ and/or STV for 6 h. Immunostaining was performed using LC3B (red) and Ate1 (green) antibodies and visualized with confocal microscopy. Nuclei (blue) were stained with Hoechst dye. **f** Quantification of autophagic vacuoles. The number of vacuoles was determined using the “Find Maxima” plug-in of the Fiji ImageJ software. *n*=10 cells. Statistical significance was assessed using a two-sided unpaired *t* test. * and # represent differences between cell types and treatments, respectively. *P≤0.05, **P≤0.01, ***P≤0.001, ****P≤0.0001, *ns*=nonsignificant. Scale bar: 10 μm

During autophagy, cytosolic LC3 (LC3-I) undergoes lipidation to form LC3-II, which is subsequently recruited into double-membrane vesicles known as autophagosomes that fuse with lysosomes [45]. To gain further insights into the contribution of Ate1 to the lysosomal degradation of cellular substrates, we monitored LC3B conversion (LC3-I to LC3-II) in the presence of the lysosomal inhibitor CQ under control (serum-rich) and/or STV conditions in *Ate1* KO cells. Initially, we observed that steady-state protein levels of the autophagic marker LC3-II were significantly higher in *Ate1* KO cells than in WT and *Ate1* KO^Ate1-1^ cells (Fig. 5a, b), suggesting that autophagic flux might be altered in Ate1-deficient cells. Time-course experiments conducted under serum-rich conditions revealed that *Ate1* KO cells displayed a reduced LC3B lipidation rate, as evidenced by the lower LC3-II/LC3-I ratio compared to WT cells (Fig. 5c). Ectopic expression of Ate1-1 in *Ate1* KO cells partially rescued this phenotype. Furthermore, exposure to STV conditions, which are known to induce autophagy [46], resulted in a twofold reduction in LC3B lipidation compared to WT cells (Fig. 5d). These observations suggest that Ate1 deficiency may block autophagosome formation, disturbing both basal and induced autophagic flux in the cell.

As the amount of LC3-II correlates with the number of autophagosomes [47], we next examined the abundance of autophagic vacuoles (autophagosomes and autolysosomes) by staining for LC3B in WT, *Ate1* KO, and *Ate1* KO^Ate1-1^ cells. Consistent with impaired autophagic flux, puncta labeled for LC3B were found to be increased in Ate1-deficient cells under basal conditions, whereas the expression of Ate1-1 partially restored it (Fig. 5e, f). Additionally, the lack of Ate1 diminished STV-induced formation of LC3B^+^ vacuoles, indicating strong autophagic suppression compared to WT cells. Taken together, these findings suggest that the formation and clearance of autophagosomes might be impaired in the absence of Ate1 and firmly support the notion that a lack of Ate1 hinders autophagy performance.

### Cells lacking Ate1 displayed an overactivated mTORC1/AMPk pathway

**Fig. 6:**
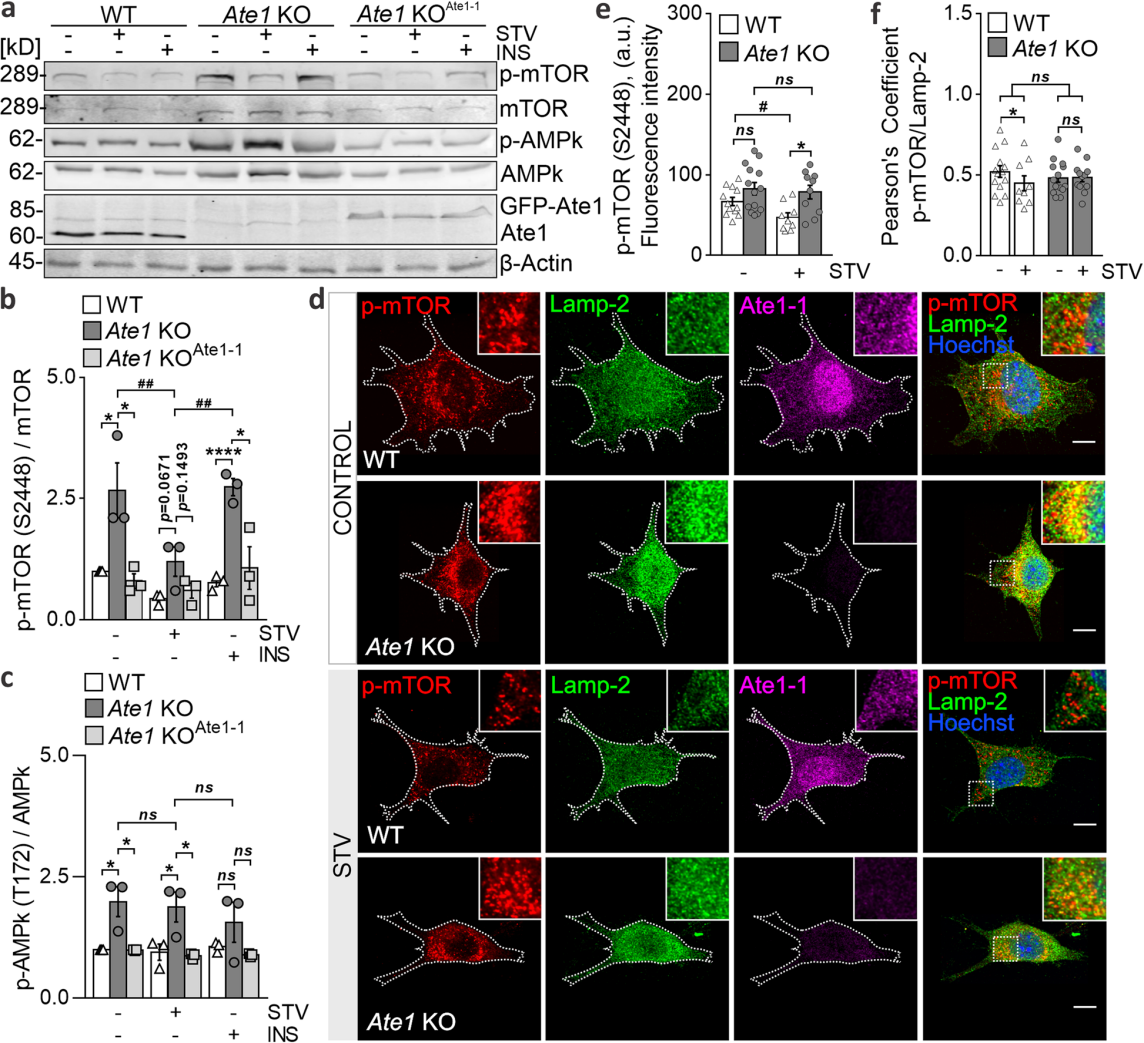
Cells lacking Ate1 displayed an overactivated mTORC1/AMPk pathway. **a** Immunoblot analysis of *p*-mTORC1 (S2448), *p*-AMPk (T172), and LC3B levels in WT, *Ate1* KO, and *Ate1* KO^Ate1-1^ cells after 6 h of STV or INS treatment. Ate1 and β-actin were used as *Ate1* deletion and loading controls, respectively. **b** Quantification of *p*-mTOR relative to total mTOR and **c** *p*-AMPk relative to total AMPk levels. Quantifications represent the mean ± SE of three independent experiments. **d** Immunofluorescence of WT and *Ate1* KO cells incubated in serum-rich (CONTROL) or STV conditions for 6 h. Immunostaining was performed using *p*-mTOR (S2448) (red), Lamp-2 (green), and Ate1 (magenta) antibodies and visualized with confocal microscopy. Nuclei (blue) were stained with Hoechst dye. Insets show higher-magnification details. **e** Quantification of total *p*-mTOR fluorescence levels. *n*=14 (CONTROL), *n*=10 (STV) *Ate1* KO cells, and *n*=14 (CONTROL), *n*=9 (STV) WT cells. **f** Pearson's correlation coefficient (PCC) between *p*-mTOR and Lamp-2 signals, calculated using the "Coloc 2" plug-in of the Fiji ImageJ software. *n*=14 (CONTROL), *n*=12 (STV) *Ate1* KO cells, and *n*=12 (CONTROL), *n*=9 (STV) WT cells. Statistical significance was assessed using a two-sided unpaired *t* test. * and # represent differences between cell types and treatments, respectively. *P≤0.05, **P≤0.01, *ns*=nonsignificant. Scale bar: 10 μm

To ascertain whether upstream signaling pathways contribute to the impaired autophagy observed in the absence of Ate1, we analyzed the activation status of mTORC1 and AMPk. Stimulation with anabolic stimuli, including insulin (INS), amino acids, and nutrients, triggers mTOR phosphorylation at serine (Ser) 2448 [48], whereas AMPk activity relies on threonine (Thr) 172 phosphorylation [49, 50]. These phosphosites, commonly used as an index of mTOR and AMPk kinase activity, were examined in WT, *Ate1* KO, and *Ate1* KO^Ate1-1^ cells after 6 h under serum-rich, STV conditions, or INS treatment by immunoblotting. In agreement with the reduced autophagic activity, Ate1-deficient cells displayed a significantly higher content of phosphorylated mTOR (*p*-mTOR) than WT cells under serum-rich conditions (Fig. 6a, b). Furthermore, STV conditions (Fig. 6a, b) or pharmacological inhibition with rapamycin (RAP) (Fig. S4) efficiently suppressed mTORC1 activity, as evidenced by the blockade of Ser2448 phosphorylation, thus revealing that this complex in *Ate1* KO cells is not refractory to nutrient cues. Conversely, INS supplementation stimulated mTORC1 phosphorylation, similar to serum-rich conditions. Remarkably, stable expression of the Ate1-1 isoform in *Ate1* KO cells reversed this overactive phenotype. Moreover, phosphorylated AMPk (*p*-AMPk) levels were higher in *Ate1* KO cells under serum-rich or STV conditions. Notably, STV stimuli did not inhibit AMPk phosphorylation, unlike mTORC1 (Fig. 6a, c), while the response to INS was similar to that of the control. Likewise, ectopic expression of Ate1-1 efficiently recovered *p*-AMPk levels to those observed in WT cells. These data demonstrate that the absence of Ate1 results in excessive phosphorylation of both mTORC1 and AMPk in response to inhibitory or activating anabolic stimuli.

Previous studies have reported that mTOR activity is linked to its subcellular localization [51]. According to the current model, sensing nutrients, energy, or glucose-dependent INS leads to the phosphorylation and translocation of the mTOR complex to lysosomes as a step required for its activation [52–54]. Consequently, we next sought to establish the intracellular distribution of mTORC1 using Lamp-2 as a lysosomal marker under serum-rich or STV conditions. In WT cells, STV conditions led to a decrease in *p*-mTOR (Fig. 6d, e), which correlated with reduced *p*-mTOR levels in lysosomes, as evidenced by the lower Pearson’s coefficient (Fig. 6d, f). However, in cells lacking Ate1, STV did not completely abrogate mTORC1 activity; instead, sustained colocalization was observed in both nutrient contexts (Fig. 6f), arguing that these lysosomes continued to carry the complex. The overphosphorylation of mTORC1 and its sustained lysosomal localization support the notion that this complex is overactivated in Ate1-deficient cells. Taken together, these results suggest that mTORC1/AMPk signaling flux is altered in cells lacking Ate1, indicating that this transferase may infer on the activation of this pathway.

## Discussion

The present findings shed light on the intricate nature of Ate1’s function, revealing a heretofore-undescribed role for this transferase as a key regulator of the autophagy pathway via modulation of the mTORC1/AMPk signaling hub. This notion is supported by multiple lines of evidence: (i) Ate1 co-clusters with p62 inclusion bodies along with substrates destined for degradation. (ii) Loss of Ate1 causes overactivation of the mTORC1/AMPk pathways, which (iii) crucially impairs autophagic flux, while autophagy induction is blunted upon serum deprivation conditions. Moreover, (iv) decreased autophagy performance in those cells leads to an accumulation of autophagic substrates, and (v) expression of the Ate1-1 isoform partially rescued the phenotype. Overall, these data suggest that Ate1-mediated arginylation may have a broader role in proteolytic mechanisms beyond its canonical role as a UPS-targeting signal.

Our study provides new insights into the subcellular distribution of Ate1 isoforms providing the first evidence of two distinct populations. A soluble and dotted pattern of each isoform was detected in the cytosol and nucleus of cells, while a second rather insoluble population of either endogenous or exogenously expressed Ate1 was detectable in the form of larger foci distributed around the periphery of the nucleus along with poly-Ub proteins when proteasome activity was compromised. A hallmark of eukaryotic cells is their compartmentalization into membraneless organelles that can serve as hubs for biomolecular condensate assembly, such as p62 bodies, harboring undegraded ubiquitin species and core autophagy-related proteins [28, 40]. In line with this, our work illustrates the ability of this transferase to assemble into supramolecular clusters containing poly-Ub proteins. These clusters are detergent-labile and thus disassemble readily upon exposure to non-ionic detergents, while the release of Ate1 from these structures is quite resistant to high salt extraction. The formation of stable macromolecular assemblies was shown to enhance multi-step cellular processes [55–57]. Indeed, coalescence of the cytosolic and membrane-embedded proteolytic machinery at few degradations hot spots along the ER membrane generates degradation microcompartments that greatly improve proteolysis efficiency [38]. Additionally, Ate1 was reported to form multi-enzyme targeting complexes with different components of the N-degron proteolytic circuit, such as E3 ubiquitin ligases, to facilitate substrates capture and degradation by the proteasome machinery [58]. Consistent with this, we demonstrate that Ate1 forms heteromeric multi-protein clusters that could be anchored to membranes rather in the cytoplasm latched onto the cytoskeleton than within them. The concomitant assembly of Ate1 into these compartments and the fact that it forms multimers that interact with many substrates [8, 11, 15, 16, 18, 22], raise the possibility that such a protein might act as a platform or as a co-scaffold protein necessary for substrate processing destined for selective autophagy when UPS processing is stalled. In this sense, the formation of inclusion bodies allows p62 to specifically select and target damaged proteins that are covered with many ubiquitin tags [44], among other degradation signals (degrons) for destruction, while sparing other materials. Zhang *et al.* findings reveal that the N-terminal arginine of arginylated proteins (N-degron), binds the ZZ domain of p62 and facilitates their removal [37, 59]. In line with this report, our study shows that endogenous Ate1 and p62 co-cluster along with its cargoes. Considering that proteasomal failure may lead to a p62-driven compensatory autophagic clearance of juxta-nuclear undegraded UPS substrates [32, 44, 60], it is therefore reasonable to postulate that Ate1 cluster and p62 body assembly could be one mechanism by which this enzyme facilitates arginylation and subsequent autophagic processing of accumulated substrates.

In strong support of this, Ate1 loss-of-function experiments demonstrate that a lack of arginylation downregulates the dynamics of autophagy under different nutritional contexts. The autophagic steady-state flux, determined by the ratio of lipidated autophagosome-recruited LC3 (LC3-II) to cytosolic LC3 (LC3-I), is lower upon an adequate supply of serum, and in fasting conditions, cells lacking Ate1 failed to foster autophagic flux. Consistent with the decline in autophagy, autophagic vacuoles and p62 bodies, which are substrates of the process being subjected to lysosomal degradation [61], were found to accumulate. Consequently, the elevated levels of LC3-II and p62 observed following Ate1 depletion would not be directly linked with cellular UPS activity but rather to disrupted autophagic regulation within Ate1-deficient cells. The expression of the Ate1-1 isoform does not completely recover the autophagic turnover rate, probably due to the fact that Ate1 is represented by multiple isoforms in these cells, thereby a full rescue might require their expression. Besides, Ate1 may be a substrate of degradation, the stability of each isoform and these findings strongly indicate that this enzyme might positively regulate the overall autophagy output in the cell. Posttranslational arginylation, in the quality control of protein and organelle autophagy is not without precedent and likely improves the fidelity of protein elimination. Certain substrates of Ate1, particularly those modified at the N-terminal residue, act as p62-activating ligands [37, 59] and stimulate ER [35] and peroxisome renewal [36] by autophagic processes known as ER-phagy and pexophagy. Our findings and the previous demonstration consistently highlight that arginylation constitutes an important posttranslational modification for cellular recycling and the maintenance of quality control.

The metabolic modulation of autophagy is mainly orchestrated by the mTORC1/AMPk pathway in response to a myriad of different stimuli [62, 63]. Consistent with an overactivated status of mTORC1, we observed that Ate1 depletion leads to an overphosphorylation of this complex and a blunted autophagic response to nutrient starvation. Likewise, Ate1-1 expression fully restores mTORC1 wild-type levels. As further evidence of mTORC1 dysregulation, the active complex that is expected to localize at the lysosomal surface in fed cells and to become cytosolic during serum-deprived conditions [51, 63, 64], remains associated with Lamp2^+^ puncta in both nutritional conditions in Ate1-deficient cells. A critical mediator of this mechanism is the p62 adaptor, which acts as a scaffold protein that interacts with Rags, Raptor and PRAS40—components of the mTOR complex—and facilitates mTORC1 lysosomal translocation and activation [33, 65]. Although it is unclear whether Ate1 interacts with p62, their distribution suggests that Ate1 might belong to a signaling complex in the same cellular compartment along with p62, as a co-scaffold protein, to potentially propagate the mTORC1/autophagy signal together with p62. While mTOR negatively regulates autophagy, the role of AMPK is more complex and highly dependent on cell types and metabolic conditions [62, 66, 67]. Here, we show that AMPk was highly activated in every nutritional condition tested in Ate1-deficient cells. Additionally, it was previously reported that *Ate1* KO MEFs exhibit an exacerbated ongoing proliferation [68], which is a high ATP-consuming mechanism and is probably responsible for the AMPk phosphorylation-activated status. Overall, the dysregulated levels of mTORC1, AMPk and p62 in Ate1-deficient cells further support the notion that Ate1 may modulate autophagy signaling through an mTORC1/AMPk-dependent mechanism.

Genetic experiments conducted in several tissues highlight the importance of Ate1 in mTOR-related metabolic processes, such as, cell migration, proliferation, and differentiation [11, 16, 68, 69]. In this regard, we recently demonstrated that silencing Ate1 in primary oligodendrocyte cultures reduces the extent and complexity of processes during differentiation and myelination [19]. *In vivo*, *Ate1* conditional KO in mouse oligodendrocytes leads to uncompacted myelin layers in spinal cord axons, a process regulated by the Akt/mTOR signaling in the central nervous system [70, 71]. Furthermore, mTORC1 is one of the main drivers of cell growth and is often dysregulated in cancer [72]. Indeed, *Ate1* KO cells were shown to exhibit a tumoral phenotype with a high proliferation rate [68]. Throughout all our and other findings, Ate1 levels were consistently found to inversely correlate with mTORC1 activity, thus highlighting the critical contribution of this enzyme in facilitating many essential cellular mechanisms. In summary, the hallmark discovery that Ate1 plays a crucial role in proteolysis, contributing to cellular protein clearance and maintaining cellular homeostasis, opened the fields of Ate1 bona-fide quality control regulator. Future investigations will determine whether translational strategies are devisable to manipulate Ate1 function, as a potential therapeutical target in the treatment of diseases, such as cancer and neurodegenerative disorders, which might contribute to restore cellular homeostasis.

## Materials and Methods

### Cell culture

*Ate1* WT, KO and KO^Ate1-1,-2,-3,-4^ MEF cell lines were kindly provided by Dr. Anna Kashina (Department of Medical Sciences, University of Pennsylvania, Philadelphia, USA). The HOG cell line was generously supplied by Dr. M. Zakin (Unité d’Expression des Genes Eucaryotes, Pasteur Institute, Paris, France). CHO-K1 cell lines were acquired from the American Type Culture Collection (A.T.C.C., Manassas, VA, USA). All cell lines were cultured in standard Dulbecco's modified eagle's medium (DMEM; Thermo Fisher Scientific, Waltham, MA, USA), which contains 4.5 g/L glucose, 4 mM L-glutamine, and 25 mmol/L Hepes. The medium was supplemented with 1 mM sodium pyruvate, 0.25 μg/mL amphotericin B (Sigma-Aldrich, St. Louis, MO, USA), 200 units/mL penicillin, 100 μg/mL streptomycin (Invitrogen, Waltham, MA, USA), and 10% fetal bovine serum (FBS) (Thermo Fisher Scientific, Waltham, MA, USA). Cells were cultured in a humidified incubator with 5% CO_2_. To maintain their authenticity, all cell lines were maintained at low passage numbers (<10) and regularly tested for mycoplasma contamination.

### Polyacrylamide gel electrophoresis (SDS-PAGE) and western blotting.

Cellular lysates were prepared in RIPA buffer (50 mM Tris-HCl pH 7.5, 150 mM NaCl, 1% (v/v) Nonidet P-40, 0.2% (p/v) sodium deoxycholate) supplemented with a cocktail of protease inhibitors (Set III, Merck Millipore, Burlington, MA, USA). For the quantification of total protein in cellular lysates, the BCA protein assay kit (Pierce Biotechnology, Waltham, MA, USA) or Bradford assay (Bio-Rad) was used. All samples were diluted with Laemmli buffer in the absence (non-reduced SDS-PAGE) or presence of 5% (v/v) β-mercaptoethanol (reduced SDS-PAGE) and heated at 95 °C for 5 min. Proteins were resolved by SDS-PAGE and transferred onto nitrocellulose membranes (GE Healthcare, Little Chalfont, UK). Transferred protein bands were visualized by staining with 0.2 % (p/v) Ponceau S in 1% (v/v) acetic acid or using a REVERT-700 total protein detection kit (LI-COR Biotechnology, Lincoln, NE, USA). Subsequently, for antigen detection with antibodies from Cell Signaling, membranes were blocked for 1 h at room temperature (RT) with 5% (p/v) bovine serum albumin (BSA) in phosphate-buffered saline (PBS) containing 0.1% Tween-20 (PBS-T). For the detection of phosphorylated protein, the membranes were blocked with 5% (p/v) BSA in Tris-buffered saline (TBS) (200 mM NaCl, 50 mM Tris-HCl pH 7.5) containing 0.1% Tween-20 (TBS-T). For the detection of Ate1, membranes were blocked with 10% (p/v) non-fat dry milk in PBS and for other proteins, membranes were blocked with 5% (p/v) non-fat dry milk. The membranes were then incubated overnight at 4 °C with the primary antibody diluted in the appropriate blocking solution, washed three times with PBS-T or TBS-T, and incubated for 1 h at RT with the secondary antibody diluted in the blocking solution. Protein bands were visualized using a high-resolution near-infrared fluorescence detection system (Odyssey Infrared Imaging System, LI-COR Biotechnology, Lincoln, NE, USA). The molecular weights of the target proteins were estimated based on known molecular weight markers run simultaneously on each gel. Densitometric analysis of immunoblots was performed using the Fiji ImageJ software.

### Antibodies

The following antibodies were used: monoclonal rat anti-Ate1 (clone 6F11, Sigma-Aldrich, St. Louis, MO, USA) which recognizes all four isoforms, monoclonal mouse anti-GFP (Roche Diagnostics, Basel, Switzerland), monoclonal mouse anti-p62 (Cell Signaling, Boston, MA, USA), polyclonal rabbit anti-p62 (MBL International Corporation, Woburn, MA, USA), polyclonal rabbit anti-LC3B (Sigma-Aldrich, St. Louis, MO, USA), monoclonal mouse anti-ubiquitin (Cytoskeleton, Denver, CO, USA), monoclonal mouse anti-polyubiquitinated proteins (Enzo Life Sciences, Doral, FL, USA), monoclonal mouse anti-vimentin (Sigma-Aldrich, St. Louis, MO, USA), monoclonal rabbit anti-mTORC1 (Cell Signaling, Boston, MA, USA), polyclonal rabbit anti-mTORC1 phosphorylated at Ser2448 (Cell Signaling, Boston, MA, USA), monoclonal rabbit anti-AMPk (Cell Signaling, Boston, MA, USA), monoclonal rabbit anti-AMPk phosphorylated at Thr172 (Cell Signaling, Boston, MA, USA), mouse monoclonal anti-γ-tubulin (Sigma-Aldrich, St. Louis, MO, USA), monoclonal mouse anti-α-tubulin (DM1A, Sigma-Aldrich, St. Louis, MO, USA), mouse monoclonal anti-β-actin (Sigma-Aldrich, St. Louis, MO, USA), mouse monoclonal anti-Gapdh (Abcam, Waltham, MA, USA), polyclonal rabbit antibody against Caveolin-1 (Abcam, Cambridge, UK), mouse monoclonal anti-lamp2 (Santa Cruz Biotechnology, Dallas, TX, USA). The secondary antibodies used for western blotting were IRDye 800CW goat anti-mouse, 680CW goat anti-rabbit antibody, 800CW goat anti-rabbit, 800CW goat anti-rat or IRDye 680CW goat anti-mouse (LI-COR Biotechnology, Lincoln, NE, USA). The secondary antibodies used for immunofluorescence were anti-rabbit Alexa Fluor 546, anti-mouse Alexa Fluor 488, anti-rat Alexa Fluor 647, anti-rat Alexa Fluor 488 or anti-mouse Alexa Fluor 630 (Invitrogen, Carlsbad, CA, USA)*.*

### Plasmids and Transfection

pEGFP-N2-Ate1-1, pEGFP-N2-Ate1-2, pEGFP-N2-Ate1-3 and pEGFP-N2-Ate1-4 plasmids were kindly provided by Dr. Anna Kashina (Department of Medical Sciences, University of Pennsylvania, Philadelphia, USA). CHO-K1 cells (A.T.C.C., Manassas, VA, USA) were transfected using polyethylenimine (Sigma-Aldrich, St Louis, MO, USA) in serum-free medium with 1 μg of the indicated plasmid per 35 mm-diameter dishes for 3 h at 37 °C. *Ate1* KO cells were transfected using Lipofectamine 2000 (Thermo Scientific, Waltham, MA, USA) and Opti-MEM (Thermo Scientific, Waltham, MA, USA) according to the manufacturer’s instructions.

### Inhibitor and inducer treatments

Inhibitors and inducers were used at the following concentrations: 20 μM chloroquine (Sigma-Aldrich, St. Louis, MO, USA), 10 μM MG132 (Sigma-Aldrich, St. Louis, MO, USA), 0.5 μM bortezomib (Selleckchem, Houston, TX, USA), 0.08 μM rapamycin (Selleckchem, Houston, TX, USA), 3.5 mg/mL insulin (Novo Nordisk, Bagsværd, Denmark), and 50 μg/mL cycloheximide (Sigma-Aldrich, St. Louis, MO, USA).

### Immunofluorescence and confocal microscopy

Cells grown on glass coverslips were washed twice with PBS and fixed with 4% (w/v) paraformaldehyde (Sigma-Aldrich, St. Louis, MO, USA) and 3% (w/v) sucrose in PBS for 20 min at RT. After three washes with PBS, the cells were permeabilized with 0.01% (w/v) Triton X-100 (TX110) (US Biological, Salem, MA, USA) in PBS for 10 min at RT. Cells were then washed with PBS and incubated for 1 h at RT with a 5% (v/v) solution of FBS or horse serum in PBS. Next, the cells were incubated with primary antibodies in a 3% (v/v) solution of FBS or horse serum in PBS. After washes with PBS, the cells were incubated for 2 h at RT with secondary antibodies diluted in a 3% (v/v) solution of FBS or horse serum in PBS. Following three washes with PBS, cell nuclei were stained with Hoechst 33258 dye (Molecular Probes, Eugene, OR, USA). The secondary antibody control is designed for and included with each experiment. The coverslips were mounted using FluorSave (Calbiochem, EMD Biosciences, San Diego, CA, USA). Confocal images were acquired using Olympus FluoView (Olympus, Center Valley, PA, USA) FV1000 and FV1200 confocal microscopes with a 60x oil immersion objective. Images (1024 x 1024 pixels) were processed using FV10-ASW and/or Fiji ImageJ software. The final images were compiled using Adobe Photoshop CC or Corel Draw 2021 software.

### Subcellular fractionation and membrane protein extraction

Cells grown in 10 cm plates were washed twice with PBS and harvested with a scraper in Tris buffer (5 mM Tris-HCl, pH 7.0) supplemented with a protease inhibitor cocktail (Set III, Merck Millipore, Burlington, MA, USA). The obtained extracts were centrifuged at 10,000 x *g* for 5 min, and the cells were resuspended in Tris-PIC buffer and vortexed every 10 min. After 30 min, the extracts were mechanically lysed by passing them through a 21-gauge needle and syringe 60 times, followed by vortexing every 10 min for an additional 30 min. The nuclear fraction and intact cells were removed by centrifugation at 500 x *g* for 5 min at 4 °C. The resulting homogenates were ultracentrifuged at 400,000 x *g* (100,000 rpm) for 1 h at 4 °C using a TLA 120.1 rotor (Beckman Coulter, Brea, CA, USA). The supernatant S_100_ and pellet P_100_ fractions were separated, and P_100_ was resuspended in Tris-PIC buffer. Proteins from each fraction were precipitated with chloroform:methanol (1:4 v/v) and analyzed by western blotting or used in subsequent experiments. Extraction of peripheral membrane proteins was performed by exposing the P fraction to 0.1 M Na_2_CO3 (Sigma-Aldrich, St. Louis, MO, USA) at a pH of 11.5 for 40 min at 4 °C. As a control, the P_100_ fractions were incubated with Tris-PIC buffer alone. Finally, the extracted proteins were separated from the remaining proteins associated with P_100_ by ultracentrifugation at 400,000 x *g* for 1 h at 4 °C. The resulting fractions were precipitated with chloroform:methanol (1:4 v/v) and analyzed by western blotting. A cell fractionation assay kit (Thermo Scientific, Waltham, MA, USA) was used for the subcellular fractionation experiments.

### Partitioning with Triton X-114

The S_100_ and P_100_ fractions obtained after subcellular fractionation were incubated with 100 μL of 5% (v/v) TX114 in Tris-PIC buffer for 1 h at 4 °C, followed by phase separation at 37 °C for 3 min. Subsequently, the samples were centrifuged at 13,000 x *g* for 1 min. The upper aqueous phase (aq) and detergent-rich lower phase (det) were separated and subjected to another partitioning round. The resulting fractions were adjusted to the same final volume and detergent content, precipitated with chloroform:methanol (1:4 v/v), and analyzed by western blotting.

### Statistics

All experiments were performed as independent biological replicates, and the n values are given in the appropriate figure legends. All statistical analyses and graphs were generated using GraphPad Prism 8.0.1. Testing was performed based on an assumed pair of samples with similar variances to address the significance of differences between samples, either one sample *t* test versus a hypothetical normalized mean of 1 for experiments where the control value was set to 1 for normalization purposes in each independent replicate, or standard Student’s *t* test in other instances. All tests were two-tailed. Statistical significance was set at *P*<0.05.

### Supplementary Information

**Additional file 1: Fig. S1.** Ate1 isoform expression and subcellular distribution. **Fig. S2**. Solubility of Ate1 in aqueous and detergent phases. **Fig. S3.** Ate1 stability and colocalization with p62. **Fig. S4.** Rapamycin effect on *p*-mTORC1 levels.

**Additional file 2: Fig. S5.** Full-length original, uncropped blots in the study.

## Data Availability

The datasets used and/or analyzed during the current study are available from the corresponding author upon reasonable request.

## Abbreviations

α-Tub: Alpha-Tubulin
β-actin: Beta-Actin
Ate1: Arginyltransferase 1
AMPk: AMP-Activated Protein kinase
BCA: Bicinchoninic Acid
BSA: Bovine Serum Albumin
BT: Bortezomib
CQ: Chloroquine
Cav1: Caveolin-1
CHO-K1: Chinese Hamster Ovary-K1
CHX: Cycloheximide
DMEM: Dulbecco's Modified Eagle's Medium
ER: Endoplasmic Reticulum
FBS: Fetal Bovine Serum
Gapdh: Glyceraldehyde-3-phosphate dehydrogenase
GFP: Green Fluorescent Protein
HOG: Human OliGodendroglioma
γ-Tub: Gamma-Tubulin
INS: Insulin
KO: Knockout
LC3B: Microtubule-Associated Protein 1A/1B-Light Chain 3B
LC3-I: cytosolic LC3
LC3-II: lipidated LC3
Lamp2: Lysosome-Associated Membrane Glycoprotein 2
mTORC1: Mammalian Target of Rapamycin Complex 1
MG132: N-(benzyloxycarbonyl)-L-leucyl-L-leucyl-L-leucinal
MEFs: Mouse Embryonic Fibroblasts
PBS: Phosphate-Buffered Saline
Poly-Ub: Poly-Ubiquitinated proteins, also referred as pUb
PTMs: Posttranslational Modifications
RAP: Rapamycin
RIPA: Radioimmunoprecipitation assay
RT: Room Temperature
SDS-PAGE: Polyacrylamide Gel Electrophoresis
Ser: Serine
STV: STarVation
SQSTM1: Sequestosome-1, also referred p62
TBS: TrisBbuffered Saline
Thr: Threonine
TX100: Triton X-100
TX114: Triton X-114
UPS: Ubiquitin Proteasome System
WL: Whole lysate
WT: Wild-Type
