# Supplementary material for: Arginyltransferase 1 modulates p62-driven autophagy via mTORC1/AMPk signaling
Source: Cell Commun Signal. 2024 Jan 31;22:87. doi: 10.1186/s12964-024-01499-9 (PMC10832197; doi:10.1186/s12964-024-01499-9)
Supplement: Supplementary file 1 — Additional file 1: Fig. S1. Ate1 isoform expression and subcellular distribution. Fig. S2. Solubility of Ate1 in aqueous and detergent phases. Fig. S3. Ate1 stability and colocalization with p62. Fig. S4. Rapamycin effect on p-mTORC1 levels. [file 12964_2024_1499_MOESM1_ESM.docx]

**Additional file 1**

**Supplementary Information**

**Fig. S1.** **(a-c)** (upper panel) Immunofluorescence of CHO‑K1 cells transfected with pEGFP‑N2‑Ate1‑2 (Ate1‑2), pEGFP‑N2‑Ate1‑3 (Ate1‑3), pEGFP‑N2‑Ate1‑4 (Ate1‑4) and incubated with 10 µM MG132 for 12 h. Immunostaining was performed and analyzed using confocal microscopy. Ate1 isoforms (green) were detected using an antibody against the GFP epitope, and poly‑Ub (pUb, red) proteins were identified using a specific antibody that did not cross-react with mono‑ubiquitinated proteins. Nuclei (blue) were stained with Hoechst dye. Pearson's correlation coefficient is expressed as r. **(a'-c')** (right panel) Insets show higher magnification of the selected regions (white dashed box). (left panel) Histogram of Ate1 isoforms and poly Ub signal intensity levels along the dashed line indicated in the inset. (lower panel) Immunoblot of Ate1 isoforms levels in CHO‑K1 cells transfected with pEGFP‑N2‑Ate1‑2 (Ate1‑2), pEGFP‑N2‑Ate1‑3 (Ate1‑3), pEGFP‑N2‑Ate1‑4 (Ate1‑4) and incubated with 10 µM MG132 at different time-points (3, 6 and 9 h). Isoform protein levels was detected using an antibody against the GFP epitope, with Gapdh as a loading control. **(d-f)** Immunoblot of MEFs *Ate1* KO^Ate1-2^, *Ate1* KO^Ate1-3^ and *Ate1* KO^Ate1-^4 cells incubated with 10 µM MG132 for 16 h. Homogenates were subjected to a subcellular fractionation process using a protein separation kit from cells in culture, according to the manufacturer's instructions. Whole lysate (WL) and extracts of cytosolic proteins (Cyt), membrane-associated proteins (Memb) from ER, Golgi, mitochondrial and plasma membrane, soluble nuclear proteins (Nuc), chromatin‑bound proteins (Chrom), cytoskeleton‑associated proteins (Cytos) were obtained. Ate1-1 levels were detected using an antibody against Ate1. Gapdh and Vimentin were used as markers for cytosolic and cytoskeleton‑associated proteins, respectively. The total protein marker was used as a loading control. Densitometric analysis was performed, and the relative Ate1 levels are provided below each lane. All immunoblot samples originate from a same experiment, and the blots were simultaneously processed. Scale bars: 10 µm (main images), 1 μm (inset).


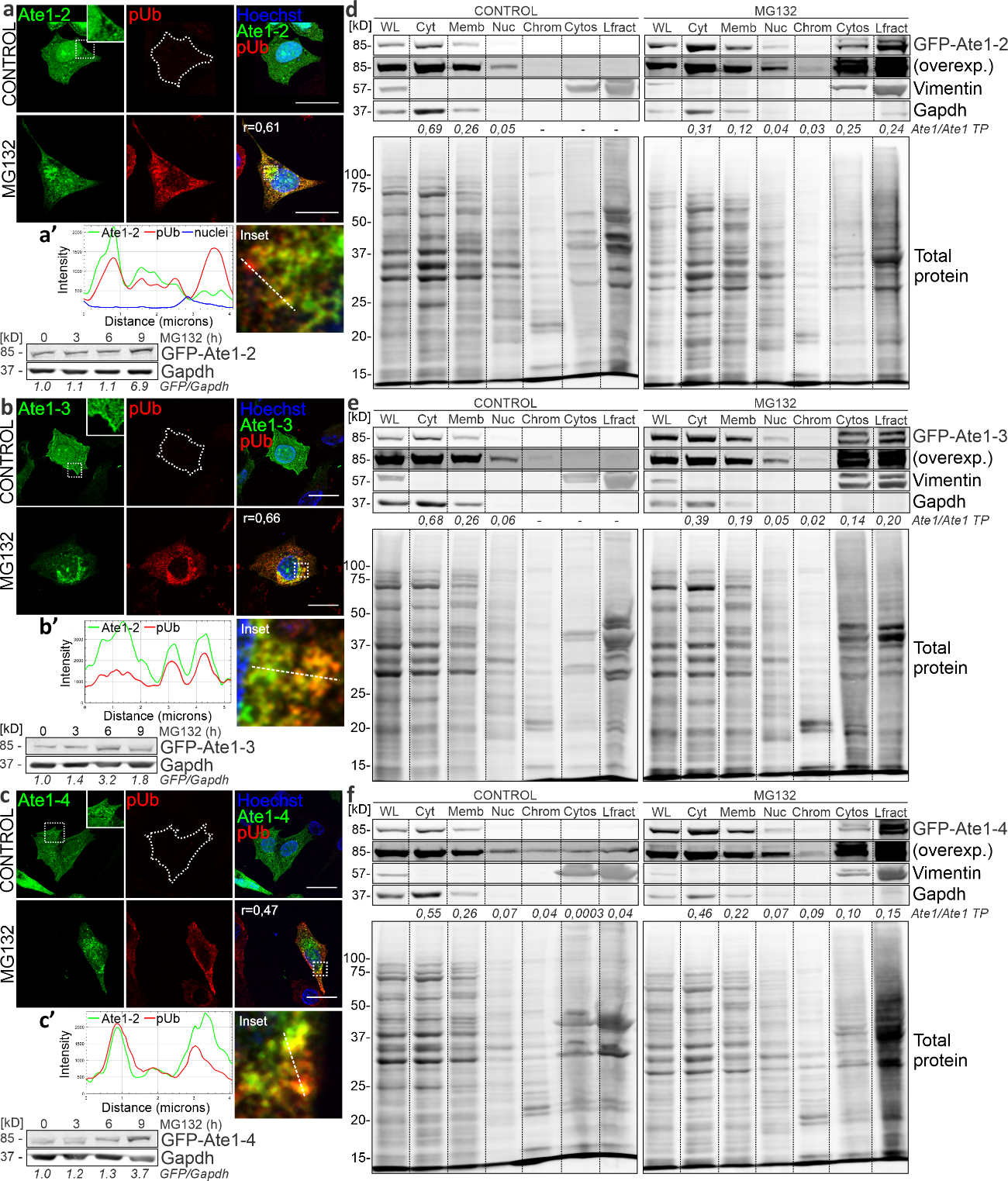


**Fig. S2. Ate1 behave as a hydrophilic protein.** Chinese hamster ovary (CHO-K1) cells were treated with 500 nM BT for 16 h. Cell lysates were subjected to ultracentrifugation at 100,000 rpm to separate them into supernatant (S_100_) and precipitate (P_100_) fractions. Cav1 and Gapdh were used as endogenous controls for membrane-bound and soluble proteins, respectively. The S_100_ and P_100_ fractions from CHO-K1 cells treated with BT, obtained by ultracentrifugation at 100,000 rpm, were incubated with the non-ionic TX114 detergent and partitioned into the aqueous (Aq) or detergent (Det) phase. The fractions were subsequently analyzed by Western blotting.


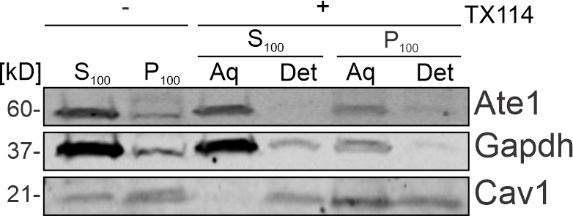


**Fig. S3. Ate1 associates with microsomal membranes in proteasome-deficient cells.** WT cells were incubated with 10 µM MG132 and/or 20 µM CQ/STV for 6 h. **(a)** Immunostaining was performed using Ate1 (green), p62 (red) and poly‑Ub (pUb, magenta) antibodies and visualized by confocal microscopy. **(b)** WT cells were incubated under serum-rich conditions for 6 h. Immunostaining was performed using Ate1 (green), LC3B (red) antibodies and visualized by confocal microscopy. (i,ii) Insets show a higher magnification of puncta where Ate1 and p62 colocalized (ii) or not (i) (white arrows). **(c)** Immunoblot of p62 levels in non-reducing and non‑heated samples. Gapdh were used as loading control. Oligo.: oligomers, Mono.: monomer. **(d)** Half‑life of Ate1 isoforms. *Ate1* KO^Ate1-1^, KO^Ate1-2^, KO^Ate1-3^, and KO^Ate1-4^ cells were incubated with 50 µg/mL CHX at different time points (2, 4, 6, 8 and 10 h). (right panel) Immunoblot of Ate1 levels. Ate1 levels were detected using an antibody against Ate1. Total protein marker was used as loading control. (left panel) Ate1 levels relative to total proteins were normalized to the control (0 h). The data were fitted to a linear regression, and the half-life (t_1/2_) was calculated from the slope: t_1/2_ = [ln(0.5)] / slope. The plotted values represent the mean ± SE. Ate1‑2, ‑3, ‑4, n=2; Ate1‑1, n=1. Statistical significance was assessed using a two‑sided unpaired *t* test. **P*≤0.05, ****P*≤0.001, *ns*=non-significant.

**
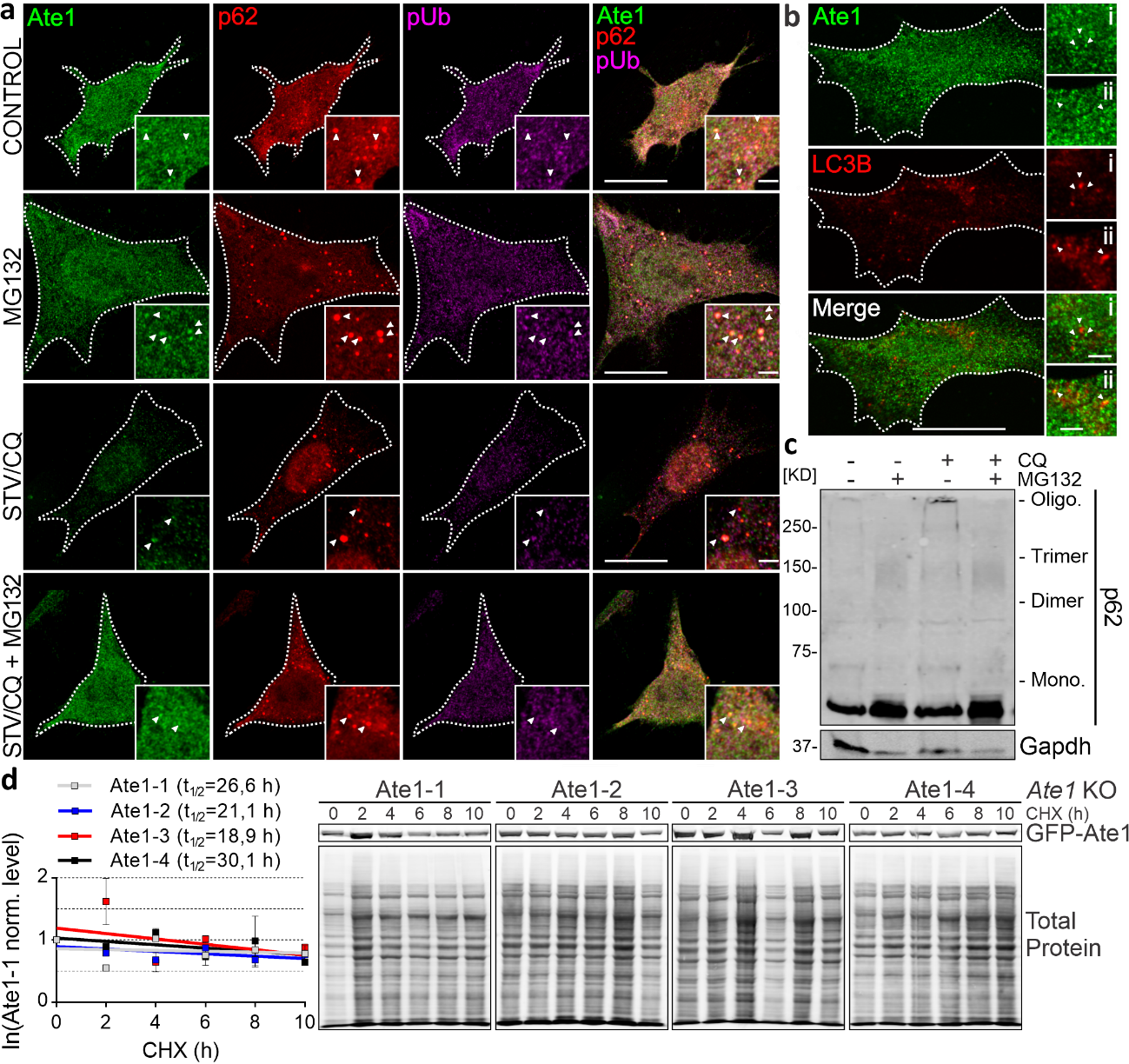
**

**Fig. S4.** **(a)** MEFs WT, *Ate1* KO and *Ate1* KO^Ate1-1^ cells were incubated with 10 nM RAP and 20 µM CQ at different time-points (0, 6 and 14 h). Samples were analyzed by Western blot using antibodies against p-mTOR (S2448), LC3B, and a total protein marker as a loading control. LC3-II quantification was expressed as the LC3‑II level relative to LC3 I. **(b)** Quantification of p-mTOR (S2448) level relative to total proteins. Quantifications are representative of two independent experiments and correspond to mean ± SE. Statistical significance was assessed using a two‑sided unpaired *t* test. **P*≤0.05, ****P*≤0.001, *ns*=nonsignificant.

**
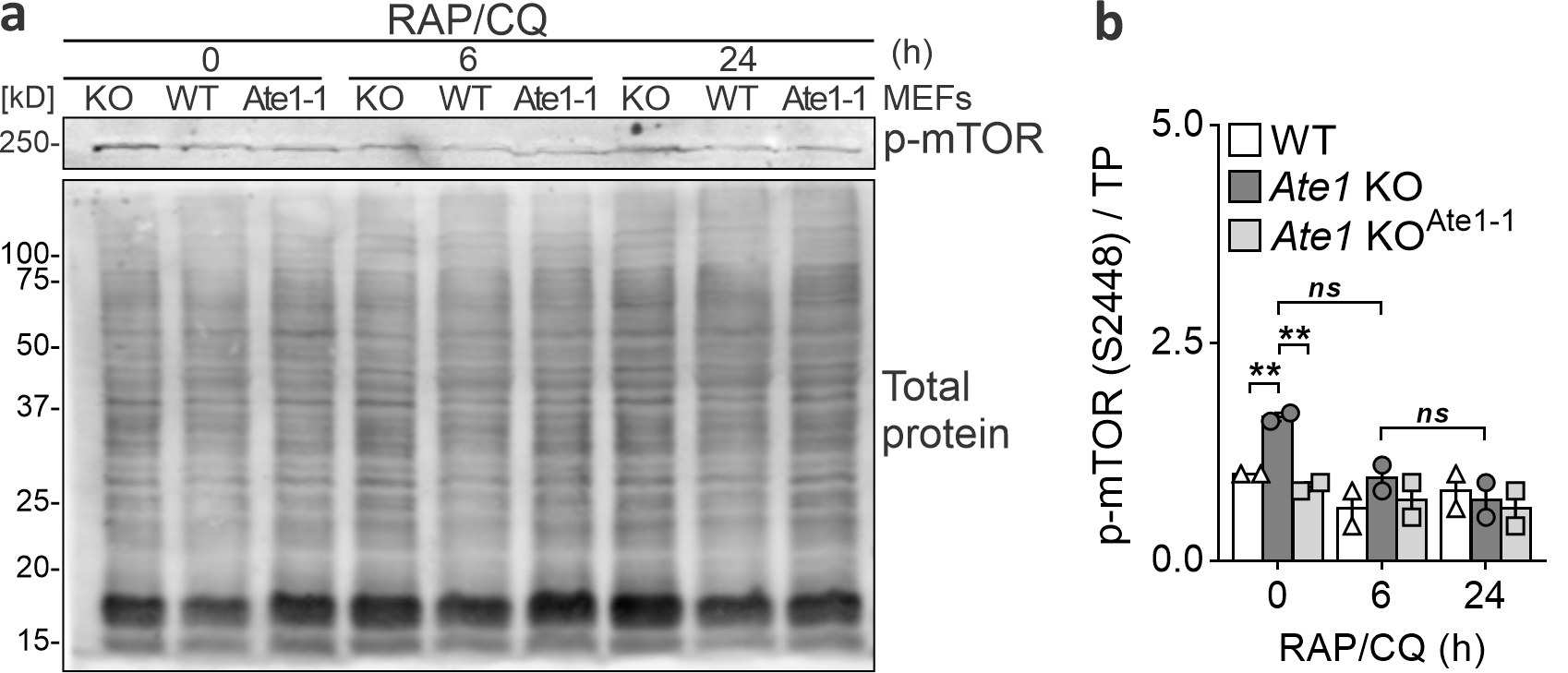
**
