# Supplementary material for: Arginyltransferase 1 modulates p62-driven autophagy via mTORC1/AMPk signaling
Source: Cell Commun Signal. 2024 Jan 31;22:87. doi: 10.1186/s12964-024-01499-9 (PMC10832197; doi:10.1186/s12964-024-01499-9)

**Additional file 2**

**Supplementary Figure Legend**

**Fig. S5.** Full-lengh original, uncropped blots in the study.

**Fig. S5
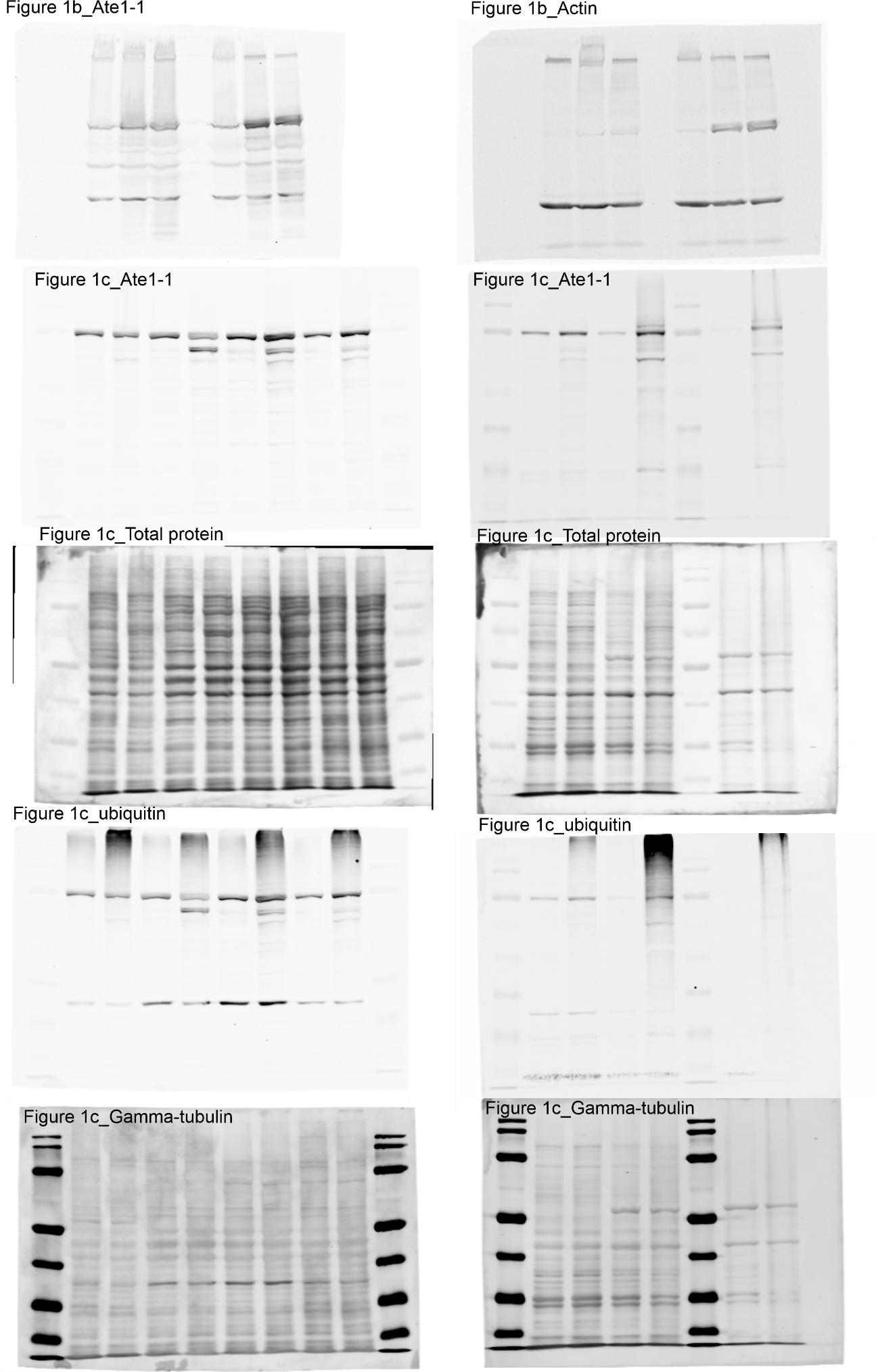
**

**
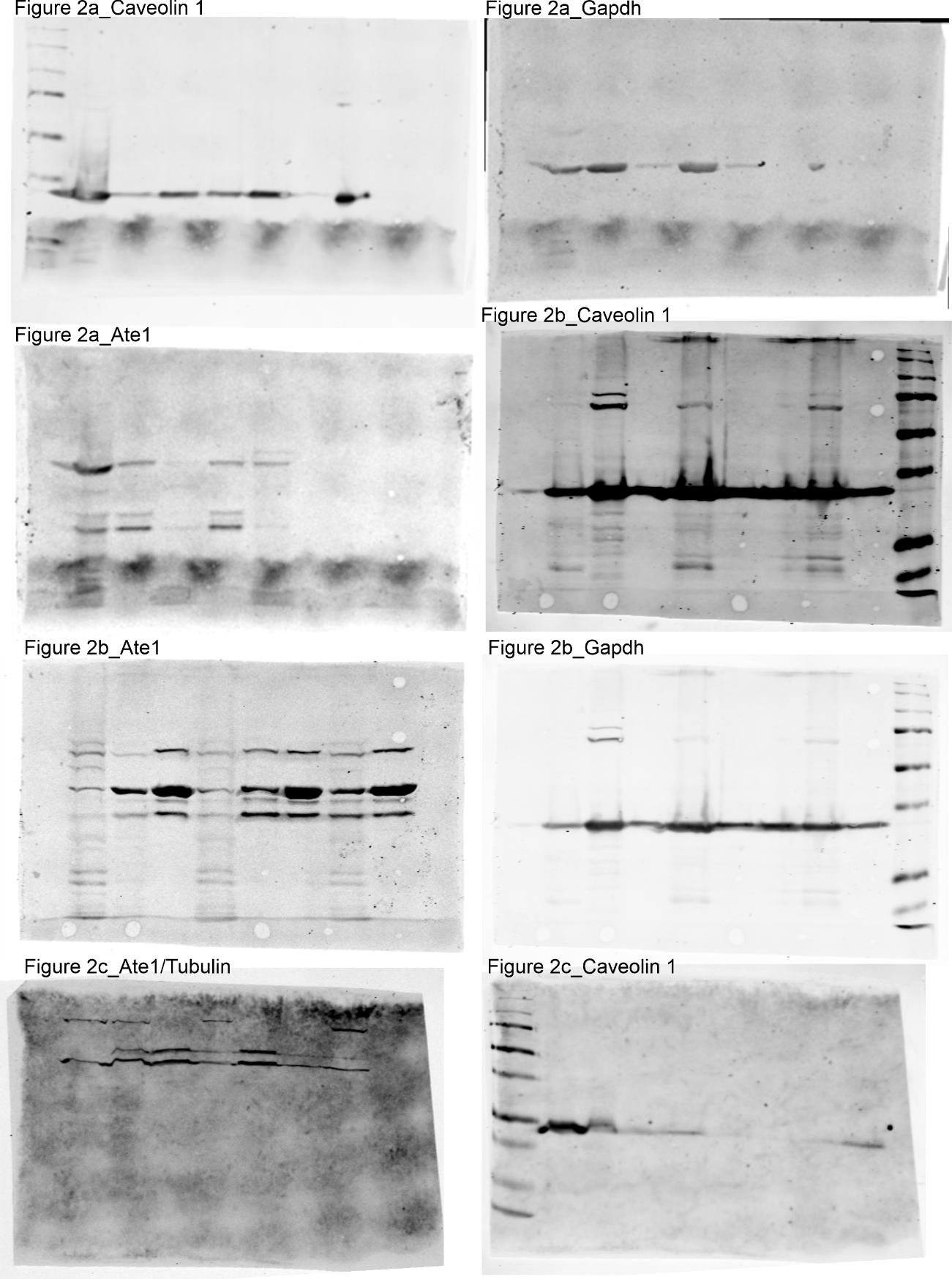
**

**
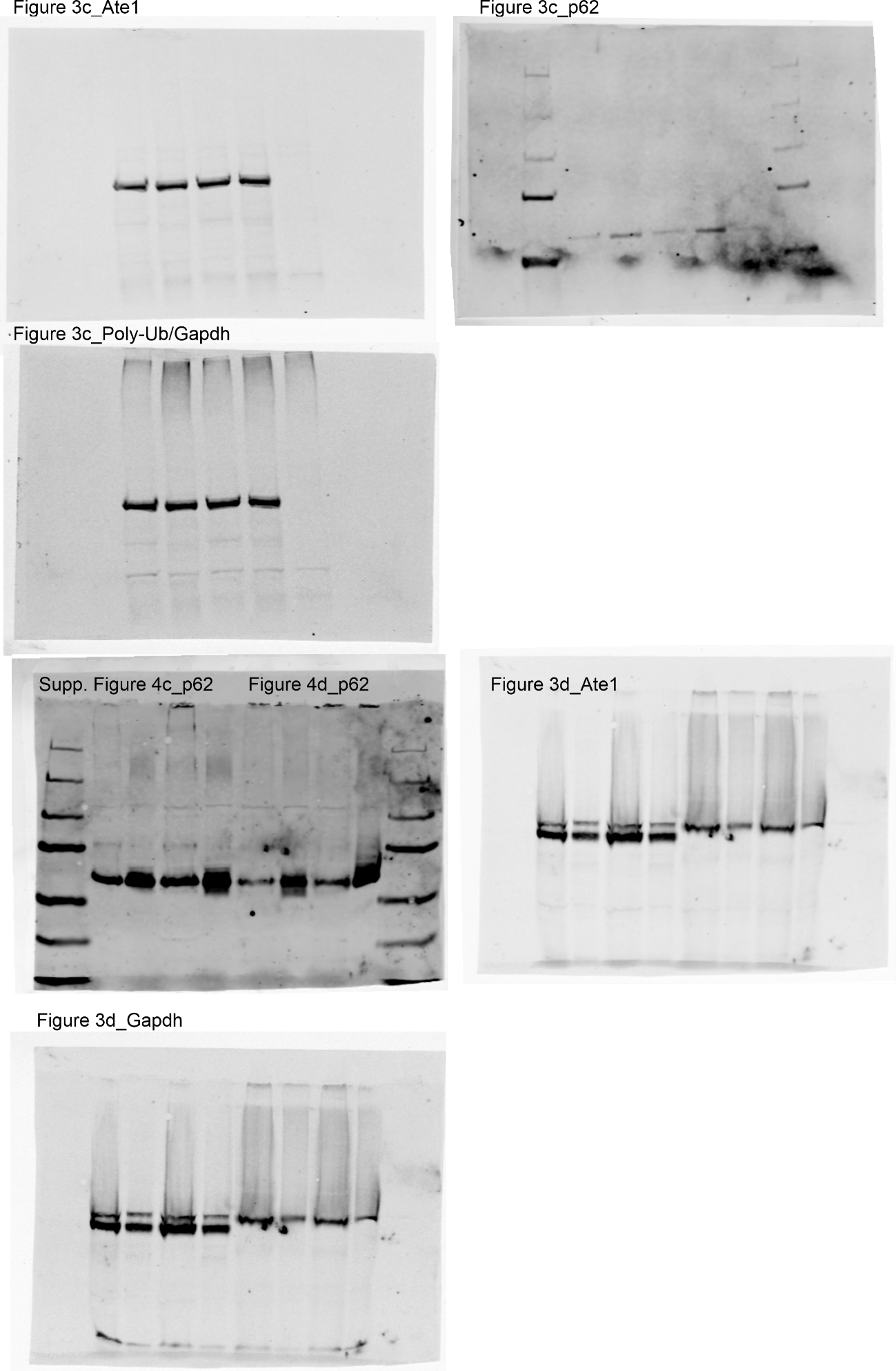
**

**
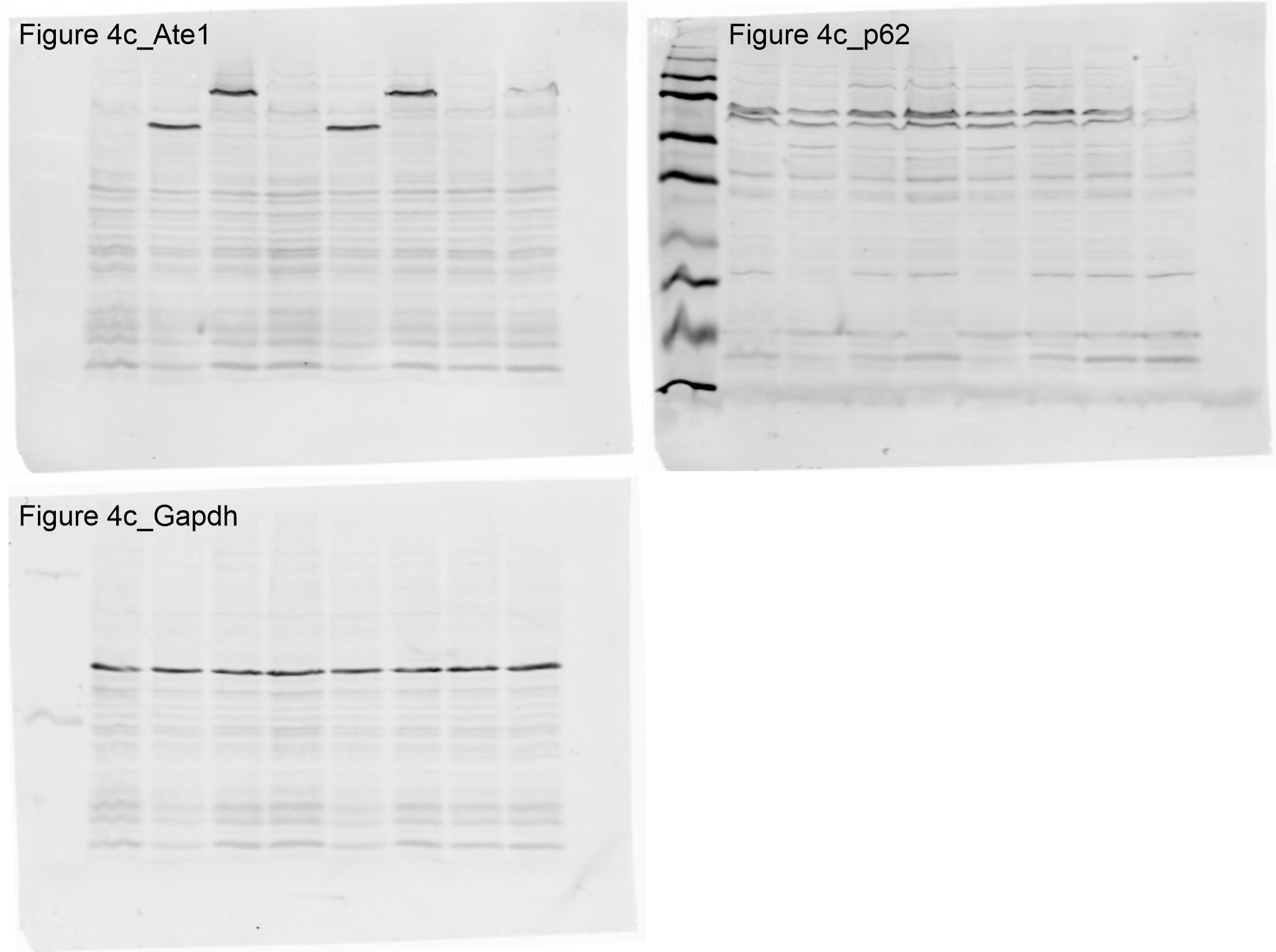
**


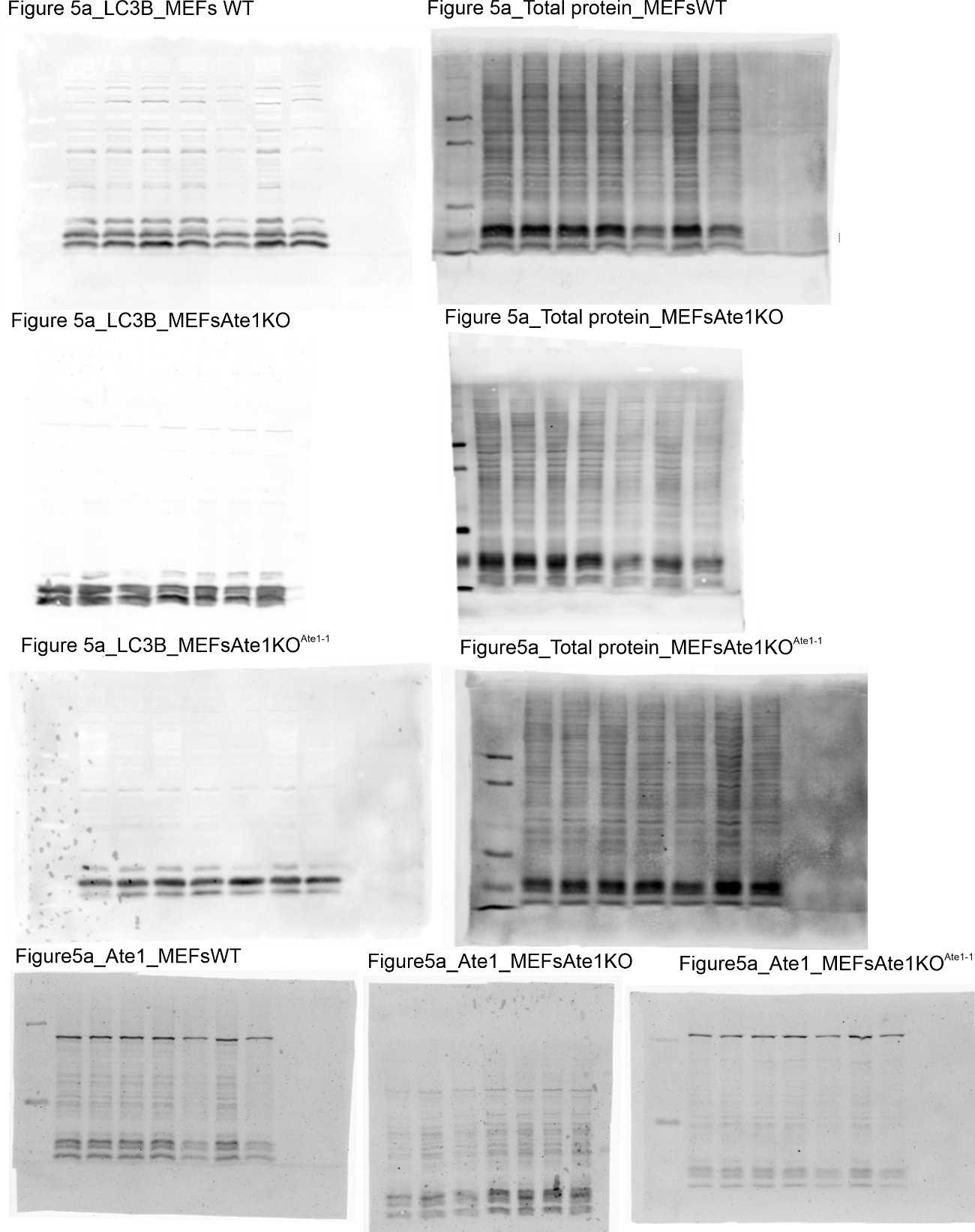


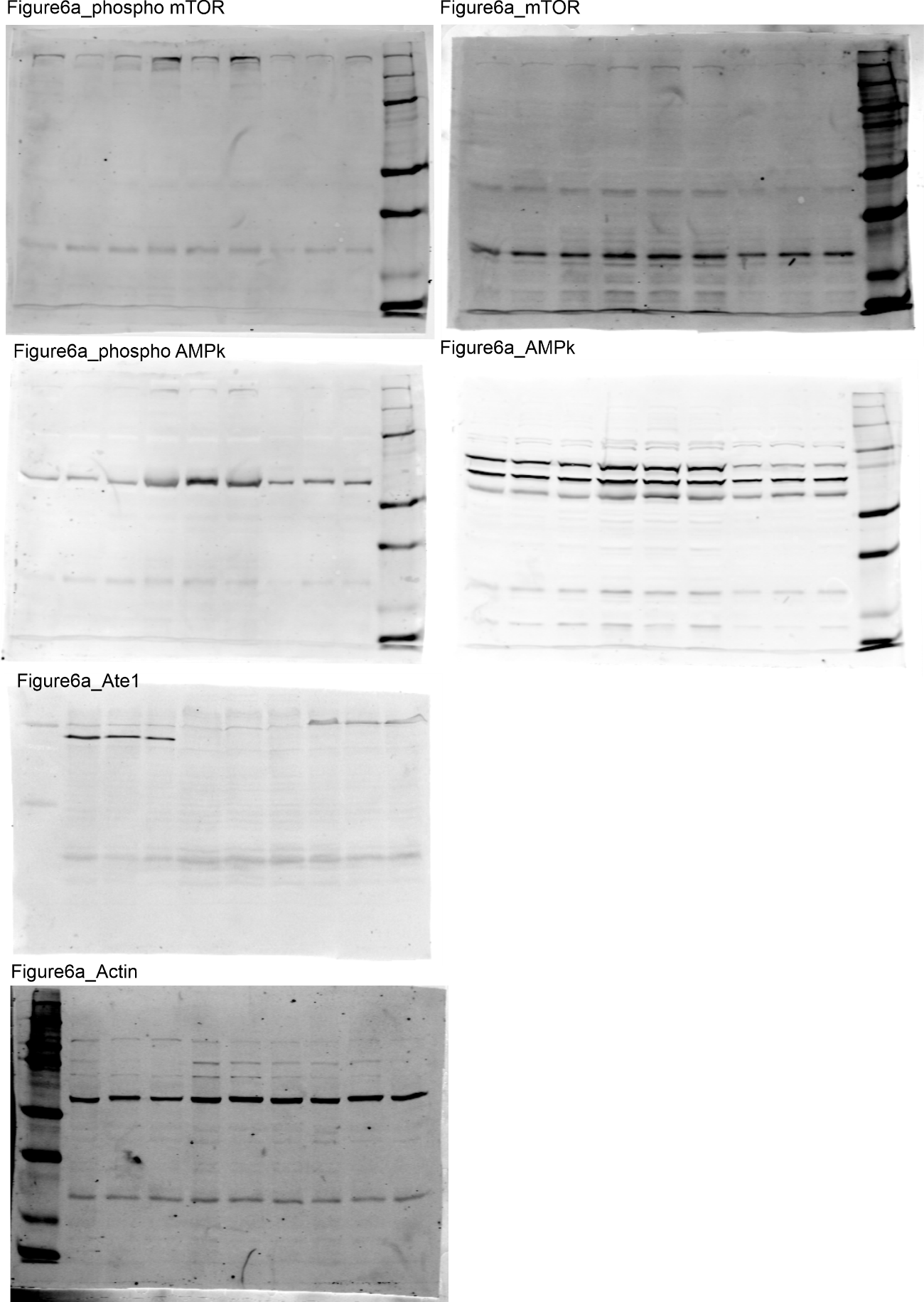

Supplement: Supplementary file 2 — Additional file 2: Fig. S5. Full-length original, uncropped blots in the study. [file 12964_2024_1499_MOESM2_ESM.docx]
